# Plasmon‐Enhanced CO_2_ Methanation over Au@Ru/TiO_2_ via Nanoscale Control of Ru Shell Thickness

**DOI:** 10.1002/anie.202518748

**Published:** 2025-11-03

**Authors:** Florian Rathmann, IbrahiM Abdelsalam, Shiqi Wang, Maja M. Kubik, Sana Frindy, Tiago V. Alves, Mykhailo Chundak, Mikko Ritala, Alexander Reznichenko, Matti Reinikainen, Pedro H. C. Camargo

**Affiliations:** ^1^ VTT Technical Research Centre of Finland P O Box 1000 Espoo FIN‐02044 Finland; ^2^ Department of Chemistry University of Helsinki A.I. Virtasen aukio 1 PO Box 55 Helsinki FIN‐0014 Finland; ^3^ Departamento de Físico‐Química Instituto de Química Universidade Federal da Bahia Rua Barão de Jeremoabo, 147 Salvador Bahia 40170‐115 Brazil

**Keywords:** CO_2_ methanation, Core–shell nanoparticles, Gold‐ruthenium bimetallic nanoparticles, Plasmonic catalysis, Visible‐light catalysis

## Abstract

Plasmonic‐catalytic nanostructures enable coupling light harvesting with chemical transformations, yet their performance critically depends on nanoscale architecture and metal‐support interactions. Here, we synthesize Au@Ru core–shell nanoparticles with tunable Ru coverage and immobilize them on TiO_2_ to create hybrid catalysts for CO_2_ methanation. By controlling Ru shell thickness, we identifyAu_60_Ru_40_/TiO_2_, featuring a thin, discontinuous shell (∼2 nm Ru nanocrystallites), as the most active composition. This catalyst combines abundant Ru active sites with preservation of the Au core's localized surface plasmon resonance (LSPR). Under 545 nm illumination, it shows a 335% rate enhancement over dark conditions at 190 °C, outperforming commercial Ru/C and remaining stable for 85 h. Optical, structural, and kinetic analysis indicate that illumination accelerates the methanation without changing the rate‐determining step, consistent with a dominant photothermal contribution. Density functional theory reveals that TiO_2_ induces strong metal‐support interactions, upshifts the Ru d‐band center, strengthens CO_2_ adsorption, and lowers the barrier for the first hydrogenation step, shifting the rate‐limiting step to CH_4_ desorption. These results establish Au@Ru/TiO_2_ as an efficient platform for visible‐light‐assisted thermocatalysis and demonstrates that nanoscale shell engineering as a generalizable strategy to optimize plasmonic catalysts for CO_2_ hydrogenation and beyond.

## Graphical abstract

Au@Ru core–shell nanoparticles on TiO_2_ integrate active Ru sites with Au plasmonic excitation. At 190 °C, 545 nm light boosts CO_2_ methanation rates by 335% while preserving >99% CH_4_ selectivity. The catalyst outperforms Ru/C and remains stable for 85 h, underscoring nanoscale shell engineering for efficient light‐assisted thermocatalysis.

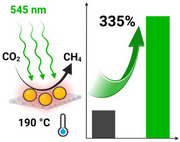

## Introduction

Transitioning to a sustainable energy system hinges on our ability to transform CO_2_ into value‐added fuels and chemicals using abundant, carbon‐neutral inputs.^[^ ^1^ ^]^ Among the most mature targets, the Sabatier reaction (CO_2_ + 4H_2_ → CH_4_ + 2H_2_O) stands out for its compatibility with existing natural‐gas infrastructure and its potential for long‐duration renewable‐energy storage.^[^ ^2^, ^3^, ^4^ ^]^ From a systems perspective, enhancing catalytic efficiency in CO_2_ methanation directly reduces the green hydrogen demandin renewable methane production, improving process economics and lowering the renewable‐electricity footprint at scale.^[^ ^5^ ^]^

Thermocatalytic CO_2_ methanation is well established over Ru, Ni, and other transition metals,^[^ ^3^ ^]^ yet relying solely on heat neglects the largely untapped potential of visible‐light harvesting.^[^ ^4^ ^]^ Plasmonic metals such as Au can convert this spectral region into intense near‐surface electromagnetic fields, generating energetic charge carriers and localized heating.^[^ ^6^, ^7^ ^]^ Coupling a plasmonic core with an active‐metal shell offers a route to combine photothermal and hot‐carrier enhancement, but realizing this potential requires precise nanoscale architectural control.^[^ ^8^ ^]^ Progress is thus hampered by three intertwined challenges. First, depositing ultrathin, conformal shells of catalytically active metals onto plasmonic templates is non‐trivial: island growth, alloying, and surface segregation can block the heterointerfaces required for efficient charge and energy transfer.^[^ ^9^, ^10^, ^11^, ^12^ ^]^ Second, even when discrete domains are formed, correlating catalytic performance with shell thickness, coverage, and crystallite size is complicated by polydispersity resulting from many synthetic protocols.^[^ ^13^, ^14^, ^15^, ^16^ ^]^ Third, the mechanistic understanding of plasmon‐enhanced methanation remains challenging due to the convolution of hot‐carrier chemistry, photothermal heating, and purely thermal routes.^[^ ^14^, ^15^, ^17^ ^]^ Fourth, although several plasmonic core–shell systems have been investigated for light‐assisted CO_2_ hydrogenation, a systematic understanding of how shell thickness and plasmonic coupling jointly influence activity and light response remains elusive.^[^ ^18^, ^19^, ^20^ ^]^ Addressing these bottlenecks demands synthetic precision combined with catalytic testing under rigorously controlled illumination.

Here, we present bimetallic Au@Ru core–shell catalysts where Ru nanocrystallites are precisely deposited onto monodisperse Au plasmonic cores, generating a library of well‐defined Ru coverages and shell thicknesses. We demonstrate that precise control of Ru shell thickness in Au@Ru/TiO_2_ enables direct correlation between nanoscale structure, plasmonic properties, and catalytic performance, providing a unified view of thermal and photothermal promotion. This architecture is designed to harness the Au core's localized surface plasmon resonance (LSPR) to drive CO_2_ methanation via the Ru shell. In this design, Au was selected as the plasmonic core because it combines exceptional chemical stability under reaction conditions with synthetic compatibility for controlled Ru shell growth. Unlike Ag, which readily oxidizes and undergoes galvanic exchange with Ru precursors, Au enables reproducible formation of discontinuous Ru shells while maintaining a strong visible range plasmon resonance that matches the excitation wavelengths used in this study. Systematic CO_2_ methanation catalytic tests under dark and visible‐light excitation revealed that intermediate Ru coverages maximize activity under dark conditions (purely thermocatalytic activation), while lower coverages exhibit the strongest light‐induced enhancements, up to a 335% rate increase at 190 °C. Under illumination, methane selectivity exceeds 99% across most conditions, with only minor CO formation at high temperatures. Our density functional theory (DFT) calculations show that Au@Ru/TiO_2_ metal‐support interactions downshift the Ru d‐band center, strengthen CO_2_ adsorption, and lower the first hydrogenation barrier relative to unsupported Au@Ru, shifting the rate‐limiting step to CH_4_ desorption. Under visible‐light excitation, the Au core's LSPR contributes primarily through a photothermal mechanism, accelerating the existing methanation pathway rather than altering its selectivity. Together, these experimental and theoretical insights establish clear structure‐performance relationships for plasmon‐mediated bimetallic catalysts and advance the broader goal of efficient, solar‐driven CO_2_‐to‐methane conversion for carbon‐neutral fuel production.

## Results and Discussion

**Scheme 1:**
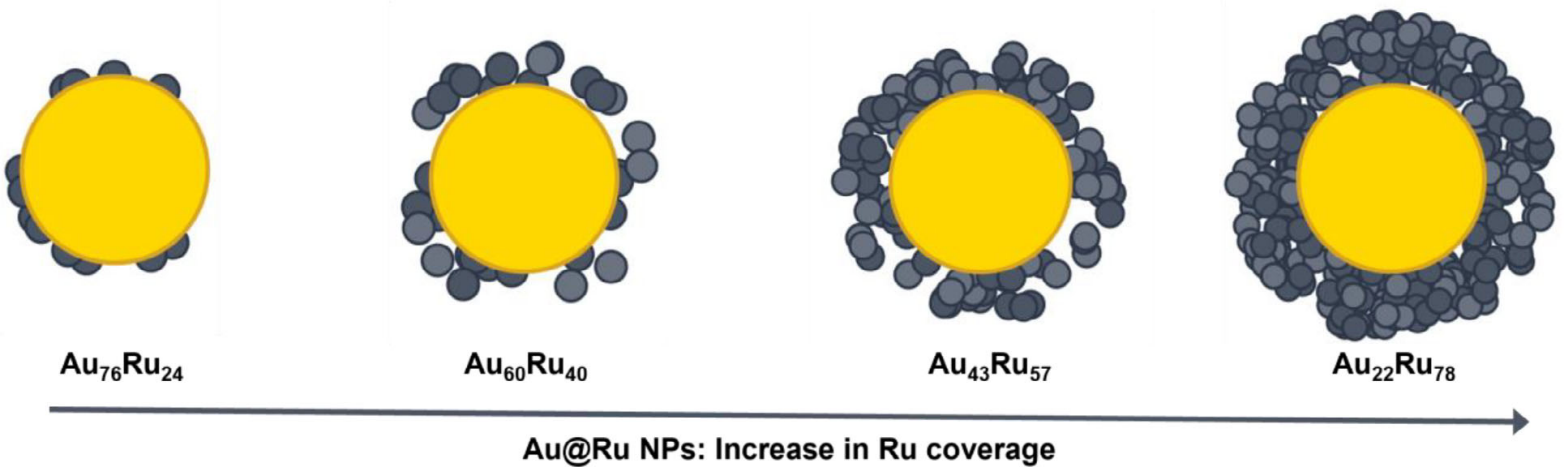
Self‐catalyzed synthesis of Au@Ru core–shell nanoparticles. RuCl_3_ is directly reduced onto plasmonic Au seeds without external reducing agents. Increasing the Ru precursor amount systematically tunes shell coverage and thickness, progressing from sparse Ru nanoclusters to a thin cluster layer and, ultimately, to a thick, dense polycrystalline shell. This morphological control preserves plasmonic activity at low–intermediate Ru loadings while increasing the density of catalytic Ru sites at higher coverages, enabling structure–performance optimization in light‐assisted CO_2_ methanation.

To explore the structure‐performance interplay in plasmon‐enhanced CO_2_ methanation, we synthesized a series of bimetallic Au@Ru core–shell nanoparticles (NPs) with tunable Ru shell thicknesses. The synthesis leveraged a self‐catalyzed reduction pathway where pre‐formed Au NPs served as both physical templates and catalytic seeds for Ru nucleation, eliminating the need for external reducing agents.^[^ ^21^ ^]^ Ru deposition is initiated by Au‐catalyzed reduction of Ru^3+^, forming the first Ru nuclei at the Au surface. These nuclei then catalyze further Ru^3+^ reduction, enabling a self‐sustaining autocatalytic growth process.^[^ ^21^ ^]^ By varying the RuCl_3_ precursor concentration during deposition, we obtained four compositions with theoretical Ru/Au atomic ratios of 0.4, 0.8, 1.6, and 4 that were designated as Au_76_Ru_24_, Au_60_Ru_40_, Au_43_Ru_57_, and Au_22_Ru_78_, respectively, based on MP‐AES analysis (Table S1). This growth protocol produced a variety of Au@Ru core‐shell morphologies (Scheme 1), from sparse Ru nanoclusters to near‐continuous polycrystalline coatings, while maintaining colloidal stability. This resulting library provides a controlled platform to correlate Ru loading, surface architecture, and interfacial design with catalytic performance under both thermal and light‐driven conditions. Although experimental procedures for nanoparticle synthesis, TiO_2_ deposition, and catalytic testing follow established methodologies, we believe the novelty of this work lies in applying precise nanoscale control of Ru shell thickness and integrated structural–optical–catalytic analysis to uncover the interplay between plasmonic excitation and interfacial Ru–TiO_2_ catalysis.

**Figure 1:**
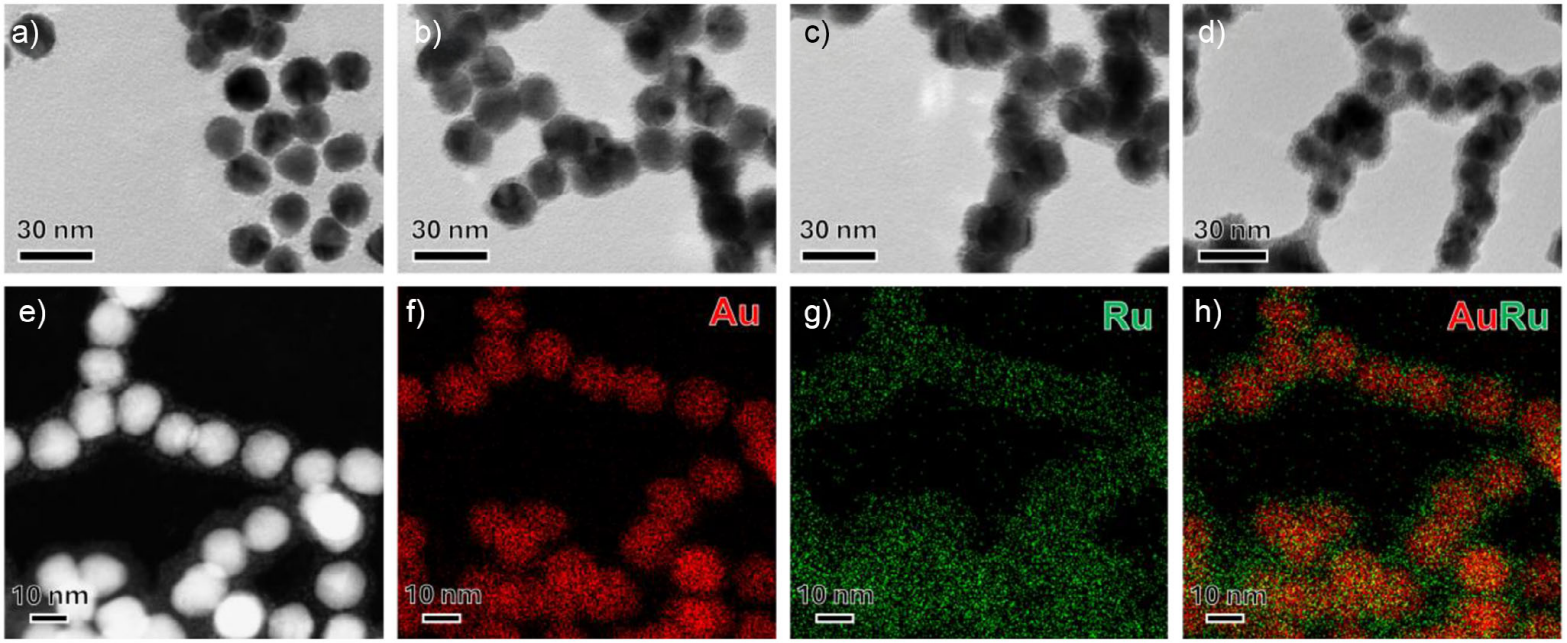
Microscopic characterization of Au@Ru core–shell nanoparticles. a–d) Representative TEM images showing the evolution of the Ru shell with increasing Ru content: a) Au_76_Ru_24_, b) Au_60_Ru_40_, c) Au_43_Ru_57_, and d) Au_22_Ru_78_. e) HAADF‐STEM image of Au_60_Ru_40_ nanoparticle highlighting Z‐contrast between the Au core and Ru shell. f–h) STEM‐EDS maps of Au_60_Ru_40_ showing the distribution of Au (f, red), Ru (g, green), and the overlay (h), confirming Ru enrichment at the periphery and a distinct core–shell architecture. Scale bars: 30 nm (a–d) and 10 nm (e–h).

Transmission electron microscopy (TEM) images revealed the morphological evolution of the Au@Ru series (Figure 1a–d). Au_76_Ru_24_ (Figure 1a) exhibits a thin, patchy shell of isolated ∼2 nm Ru clusters on the Au surface. As the Ru/Au ratio increases, the shell progressively transitions from discrete clusters (Figure 1b,c) to a near‐continuous, high‐contrast coating in Au_22_Ru_78_ (Figure 1d), consistent with dense Ru coverage. All samples retain a spherical core–shell architecture with the core having an average size of 14.5 ± 1 nm, the shell contrast and thickness increasing with Ru content, with Ru clusters averaging ∼2 nm in Au_76_Ru_24_ that coalesce into a continuous shell up to ∼7 nm in thickness in Au_22_Ru_78_. High‐angle annular dark‐field scanning transmission electron microscopy (HAADF‐STEM) imaging of Au_60_Ru_40_ (Figure 1e) shows a bright Au core surrounded by a lower‐contrast shell, reflecting the atomic number difference between Au and Ru. STEM–energy dispersive X‐ray spectroscopy (EDS) mapping of the same particle (Figure 1f–h) confirms that Au is confined to the core, while Ru is enriched at the periphery in a shell‐like distribution. The absence of detectable alloying or intermixing at this resolution underscores that the self‐catalyzed deposition protocol achieves precise Ru spatial localization and tunable shell coverage while preserving structural integrity across the series.

**Figure 2:**
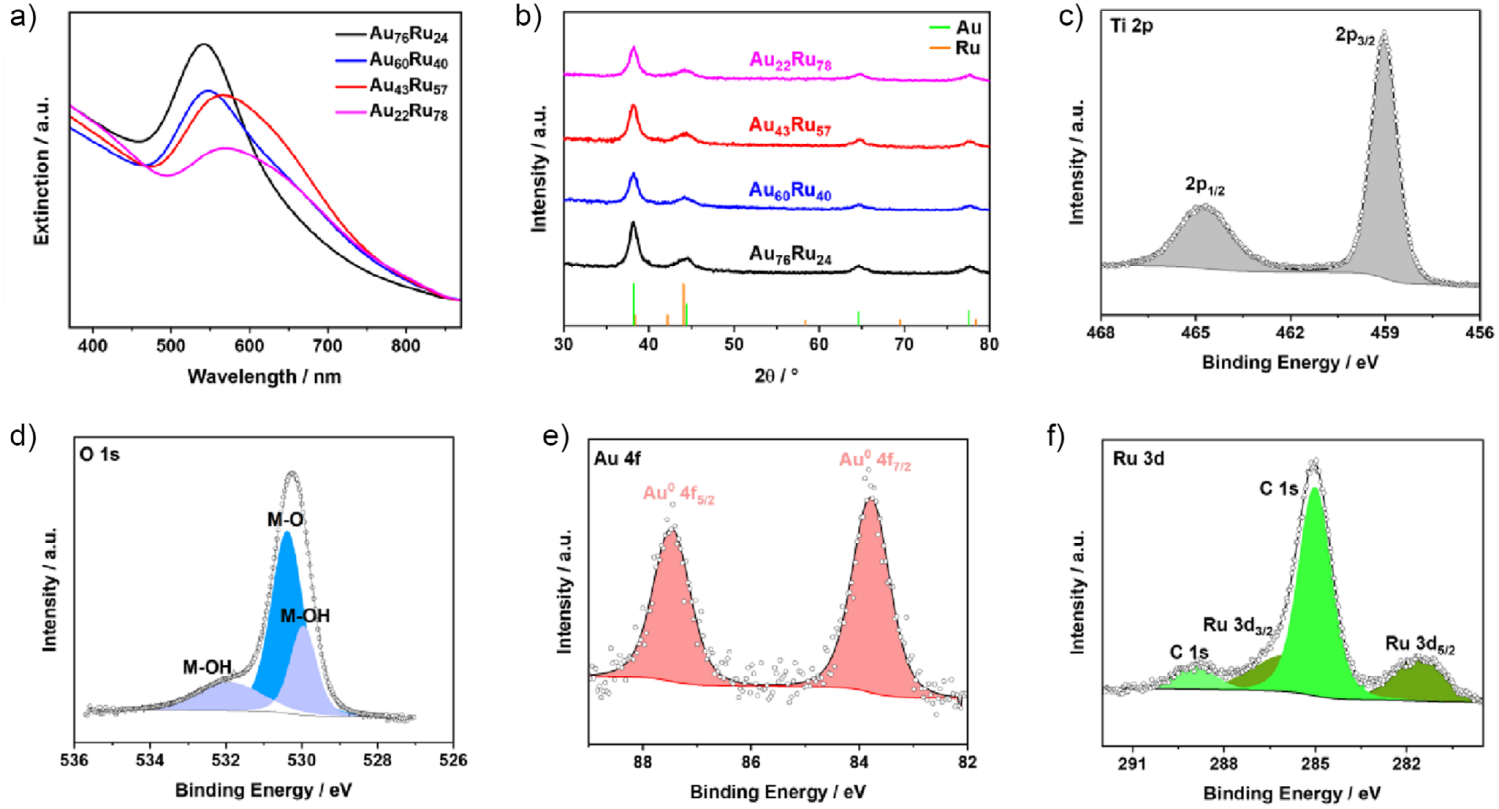
Spectroscopic and structural characterization of Au@Ru nanoparticles. a) UV–vis extinction spectra showing LSPR redshift and attenuation with increasing Ru coverage: Au_76_Ru_24_ (black), Au_60_Ru_40_ (blue), Au_43_Ru_57_ (red), and Au_22_Ru_78_ (magenta). b) XRD patterns displaying fcc Au reflections (JCPDS No. 04‐0784) with no Ru peaks, consistent with sub‐2 nm Ru clusters observed by TEM. C–F) XPS core‐level spectra of Au_60_Ru_40_/TiO_2_: c) Ti 2p confirming Ti^4+^ in TiO_2_; d) O 1s showing lattice O^2−^ and surface hydroxyls; e) Au 4f indicating metallic Au^0^; f) Ru 3d revealing metallic Ru^0^ and partially oxidized Ru^δ⁺^ species. Together, these data confirm a core–shell architecture with a plasmonically active Au core encapsulated by a thin, partially oxidized Ru shell.

The optical and crystalline evolution of the Au@Ru series was probed by UV–visible spectroscopy and powder X‐ray diffraction (XRD). In the UV–vis spectra (Figure 2a), the LSPR band of Au NPs (∼525 nm) exhibits a systematic redshift and attenuation with increasing Ru loading.^[^ ^22^, ^23^ ^]^ Au_76_Ru_24_ retains a pronounced, slightly broadened plasmonic peak, consistent with partial surface coverage by Ru nanoclusters. With higher Ru content (Au_60_Ru_40_ and Au_43_Ru_57_), the LSPR feature progressively broadens and diminishes, reflecting increased dielectric screening and light scattering from the growing Ru shell. In Au_22_Ru_78_, the plasmonic peak is almost fully suppressed due to “plasmonic shielding” by the dense Ru coating.^[^ ^24^, ^25^ ^]^ These observations confirm that Ru coverage can finely tune the nanostructure's optical response, a key parameter for light–matter interactions in plasmon‐enhanced catalysis. To complement the experimental extinction spectra, discrete dipole approximation (DDA) simulations were performed to evaluate the influence of Ru shell thickness on the plasmonic response (Figure S3). The calculated near‐field distributions at 545 nm (wavelength employed in our catalytic investigations) reveal a progressive attenuation of the localized electromagnetic field as the Ru coverage increases. The maximum field enhancement (|E|2/|E_0_|2) decreases from 29.5 for bare Au to 25.8, 12.1, and 8.2 for Au@Ru with Ru shell thicknesses of 2, 4, and 7 nm, respectively. These results mirror the experimental UV–vis trends (Figure 2a), in which the Au plasmon resonance redshifts and dampens with increasing Ru content. The simulations confirm that thin Ru shells preserve strong plasmonic coupling, whereas thicker Ru coatings increasingly screen the Au core and attenuate the local electromagnetic field.

XRD patterns (Figure 2b) of all samples display dominant reflections of face‐centered cubic (fcc) metallic Au (JCPDS No. 04‐0784), with peaks at 38.2°, 44.4°, and 64.6° assigned to the (111), (200), and (220) planes. No distinct Ru reflections are visible, consistent with the presence of 2–7 nm Ru clusters at the Au surface as observed in TEM.^[^ ^24^, ^26^ ^]^ The absence of new alloy phases or significant Au peak shifts confirms that Au and Ru remain phase‐separated in a core–shell configuration across the series.

To evaluate their catalytic properties in gas‐phase CO_2_ methanation, the colloidal Au@Ru nanoparticles were immobilized onto a commercial TiO_2_ support to form Au@Ru/TiO_2_ hybrids. TiO_2_ was chosen for its high surface area, thermal stability, and proven compatibility with plasmonic and catalytic systems.^[^ ^27^, ^28^, ^29^ ^]^ Immobilization was performed via an impregnation–drying method that preserved the structural integrity of the core–shell nanoparticles. TEM images of the supported catalysts (Figure S4) confirm uniform dispersion of Au@Ru particles across the TiO_2_ surface, with no detectable agglomeration, sintering, or morphological degradation. This demonstrates that the synthetic protocol yields supported catalysts with retained nanoarchitecture, suitable for subsequent thermal and plasmonic performance testing. The experimentally determined (MP‐AES) metal loadings for Au_76_Ru_24_, Au_60_Ru_40_, Au_43_Ru_57_, and Au_22_Ru_78_ were 3.1, 3.4, 4.1, and 6.5 wt %, respectively. This corresponded to a Ru loading of 0.4, 0.9, 1.7, and 4.3 wt %, respectively (Table S1).

X‐ray photoelectron spectroscopy (XPS) was used to probe the surface composition and electronic states of the supported Au_60_Ru_40_/TiO_2_ catalysts (Figure 2c–f). The Ti 2p spectrum (Figure 2c) displays the Ti^4+^ doublet with the Ti 2p_3/2_ peak at 458.7 eV, confirming that the anatase lattice is preserved. The O 1s region (Figure 2d) consists of lattice O^2−^ at 529.9 eV and a higher‐binding‐energy shoulder at ∼531.4 eV from surface hydroxyls, indicating a moderately hydroxylated TiO_2_ surface.^[^ ^30^ ^]^ The Au 4f spectrum (Figure 2e) is dominated by metallic Au^0^ peaks (4f_7/2 _ = 83.9 eV; 4f_5/2_ = 87.6 eV), showing that the Au core remains unoxidized, critical for maintaining plasmonic activity.^[^ ^31^, ^32^ ^]^ In the Ru 3d region (Figure 2f), the C 1s peak at 284.8 eV overlaps with Ru 3d_3/2_, but deconvolution of Ru 3d_5/2_ reveals components at 280.2 eV (Ru^0^) and 281.6 eV (Ru^δ⁺^, e.g., RuO_2_).^[^ ^29^, ^33^ ^]^ The oxidized fraction (∼35%) suggests partial surface oxidation of ultrasmall Ru domains upon air exposure. The coexistence of Ru^0^ and Ru^δ⁺^ indicates a chemically heterogeneous shell composed of Ru nanocrystallites with partially oxidized surfaces. The surface Ru/Au atomic ratio from XPS (0.8) slightly exceeds the bulk ratio (0.7), consistent with a thin Ru shell enriched at the nanoparticle surface. These XPS results, in agreement with HAADF‐STEM, EDS mapping, and XRD results, consistently demonstrate a core–shell architecture where a plasmonically active Au core is encapsulated by a thin, partially oxidized Ru shell, an arrangement designed to couple efficient light harvesting with abundant catalytic sites.

To assess active‐site availability and CO_2_ adsorption characteristics, pulsed H_2_ chemisorption, static CO_2_ chemisorption, and CO_2_ temperature‐programmed desorption (TPD) measurements were performed on Au_60_Ru_40_/TiO_2_ (Figure S5A–C). Pulsed H_2_ chemisorption at 35 °C (Figure S5A) gave a total uptake of 7.6 µmol g_cat_^−1^. Assuming that one hydrogen atom is adsorbed on each Ru surface atom, this corresponds to 15.1 µmol surface Ru atoms per gram of catalyst. Relative to the theoretical total Ru content (109 µmol g_cat_^−1^, based on 1.1 wt % Ru loading), the estimated dispersion is 14%. This value likely represents an upper bound, as hydrogen spillover onto TiO_2_ can inflate the measured uptake, while strong metal–support interactions may partially encapsulate Ru particles and reduce accessibility.^[^ ^34^, ^35^, ^36^ ^]^

Static CO_2_ chemisorption at 35 °C (Figure S5B) yielded an initial uptake of 137 µmol g_cat_^−1^. A second cycle gave 128 µmol g_cat_^−1^, indicating a strongly chemisorbed fraction of 9 µmol g_cat_^−1^. This corresponds to ∼58% of the H_2_‐accessible Ru sites, suggesting that a substantial portion of surface Ru atoms can also interact with CO_2_ under the reaction conditions. The differing magnitudes of adsorption reflect the distinct adsorption modes and energetics of H_2_ and CO_2_. For comparison, bare TiO_2_ was evaluated under identical conditions (Figure S5D). The CO_2_ adsorption isotherms of bare TiO_2_ (Figure S5D) exhibited first and second cycle intercepts of 140 and 137 µmol g_cat_^−1^, respectively, corresponding to a chemisorbed fraction of only 2 µmol g_cat_^−1^. This value is significantly lower than that of Au_60_Ru_40_/TiO_2_ (9 µmol g_cat_^−1^; Figure S5B), confirming that the strongly retained CO_2_ originates from the metal–support catalyst rather than from the TiO_2_ support itself.

CO_2_ TPD profiles (Figure S5C) revealed two distinct desorption regions: a low‐temperature feature centered around 130 °C and a broader high‐temperature feature initiating near 380 °C and peaking at ∼460 °C. These are consistent with CO_2_ bound to weaker and stronger basic sites, respectively, as reported for TiO_2_‐based catalysts.^[^ ^37^ ^]^ Only the low‐temperature species are likely to participate in hydrogenation at the 190 °C reaction temperature; the high‐temperature fraction is likely inert under the operating conditions.^[^ ^38^ ^]^ The corresponding CO_2_ TPD profile of bare TiO_2_ (Figure S5E) displayed desorption peaks centered at ∼70 °C and a broader composite feature with maxima at ∼370 and ∼470 °C, consistent with the weak‐to‐moderate adsorption sites typically observed for TiO_2_‐based oxides. In contrast, Au_60_Ru_40_/TiO_2_ exhibited sharper, higher‐intensity features extending to higher temperature, indicating stronger CO_2_ binding associated with Ru domains and Ru–TiO_2_ interfacial sites.

Overall, the combined H_2_ and CO_2_ chemisorption/TPD results indicate that Au_60_Ru_40_/TiO_2_ offers a balanced population of accessible Ru sites for both H_2_ activation and CO_2_ binding. This interplay of site availability and binding strength is likely a key factor in enabling high dark‐state methanation rates and pronounced light‐induced activity enhancements.

With the surface composition and oxidation states established, we evaluated CO_2_ methanation performance under both thermal and plasmonic excitation. Prior to testing, all catalysts underwent in situ H_2_ pretreatment at elevated temperature to fully reduce surface RuO_2_ to metallic Ru, ensuring that turnover occurred exclusively on metallic Ru sites. This step was particularly important given the XPS evidence for partial oxidation of the thin Ru shell upon air exposure. The pretreatment restores the metallic character of Ru while preserving the underlying Au core, allowing a direct comparison of intrinsic thermal activity with plasmon‐enhanced performance. Catalytic measurements were conducted under two conditions: (i) dark, thermally driven conditions probing the Ru shell's baseline activity, and (ii) visible‐light illumination to activate the plasmonic Au core and introduce hot‐carrier and photothermal contributions.

**Figure 3:**
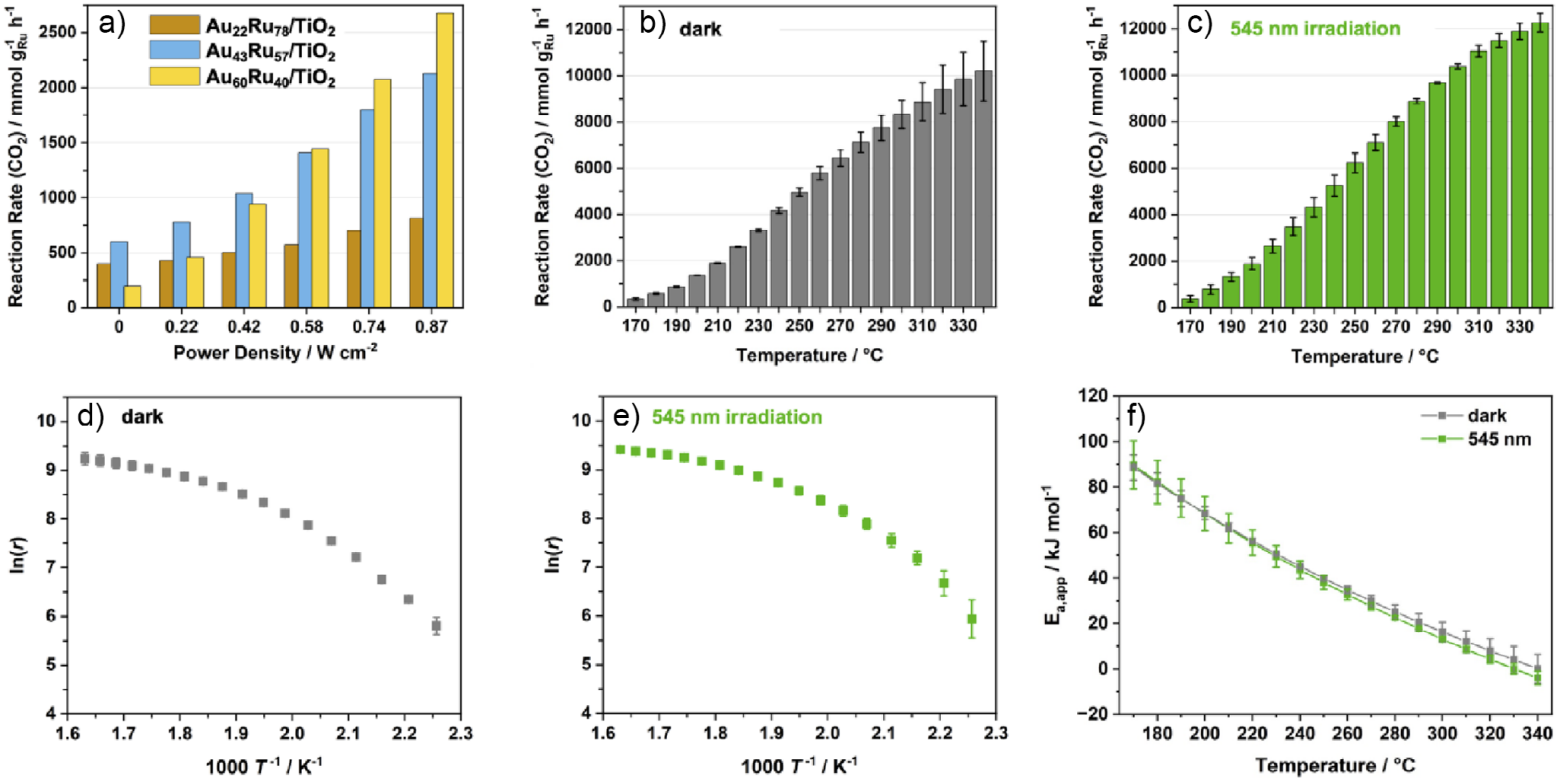
Catalytic performance and kinetic analysis of Au@Ru/TiO_2_ in CO_2_ methanation. a) Screening of Au_60_Ru_40_, Au_43_Ru_57_, and Au_22_Ru_78_ under dark and 545 nm illumination at varying power densities (190 °C). b, c) Temperature‐dependent rates for Au_60_Ru_40_/TiO_2_ under B) dark and C) illuminated conditions. d, e) Arrhenius plots from (B, C) displaying curvature indicative of complex surface kinetics. f) Temperature‐dependent apparent activation energies (*E*_a,app_) derived from (D, E) for dark (gray) and illuminated (green) conditions, revealing similar profiles and indicating that illumination accelerates the existing pathway without changing the rate‐determining step.

Initial screening of TiO_2_‐supported catalysts (Figure 3a) included Au_60_Ru_40_, Au_43_Ru_57_, and Au_22_Ru_78_. Au_76_Ru_24_ was excluded due to its observed very low activity likely arising from insufficient Ru coverage. Under the dark conditions (0 W cm^−2^), Au_43_Ru_57_ delivered the highest rate, followed by Au_22_Ru_78_ and Au_60_Ru_40_, indicating that intermediate Ru coverages provide an optimal balance of active‐site density and accessibility for thermal methanation.^[^ ^29^, ^39^ ^]^ Upon illumination, however, the trend changed: Au_60_Ru_40_ progressively exhibited the strongest light‐induced enhancement, surpassing the other catalysts at moderate power densities and emerging as the most active composition at high intensities. This pronounced light response suggests that the thinner Ru shell in Au_60_Ru_40_ maintains strong plasmonic coupling between the Au core and catalytic surface, enabling a synergistic combination of thermal, photothermal, and hot‐carrier effects.^[^ ^22^ ^]^ This is in agreement with UV–vis data and DDA simulations where a thinner Ru shell exhibits the strongest light‐induced enhancement. The systematic variation of Ru shell thickness in Au@Ru/TiO_2_ establishes a direct structure–performance relationship, revealing an optimal regime (Au_60_Ru_40_/TiO_2_) where strong Au plasmon coupling and sufficient Ru coverage coexist. This tunability, together with the clear mechanistic differentiation between photothermal and hot‐carrier effects, distinguishes the present work from previous Au@metal plasmonic catalysts reported for CO_2_ methanation. Based on these results, Au_60_Ru_40_/TiO_2_ was selected for detailed catalytic and mechanistic studies.

The temperature‐dependent CO_2_ methanation performance of Au_60_Ru_40_/TiO_2_ was evaluated under dark and illuminated conditions (545 nm, 0.67 W cm^−^^2^) from 170 to 340 °C in 10 °C increments (Figure 3b,c) from the same catalyst batch (full dataset in described Table S2). In both cases, the reaction rate increased steadily with temperature, consistent with thermally activated CO_2_ methanation.^[^ ^40^ ^]^ Under dark conditions, the rate increased from 332 mmol g_Ru_^−1^ h^−1^ at 170 °C to 10 200 mmol g_Ru_^−1^ h^−1^ at 340 °C. Upon illumination, the rate at 170 °C increased to 202 mmol g_Ru_^−1^ h^−1^ (+14% relative to dark), with the relative enhancement peaking at + 53% near 190 °C before declining at higher temperatures. At 340 °C, the illuminated rate reached 12 260 mmol g_Ru_^−1^ h^−1^ (+20%), suggesting that the plasmonic contribution becomes less significant once thermal energy alone can drive the reaction near kinetic or thermodynamic limits.

To further assess this trend, additional tests at 340 and 400 °C were performed on the same catalyst batch under both regimes. At 340 °C, the dark and illuminated rates were 10 300 and 10 900 mmol g_Ru_^−1^ h^−1^, respectively (+6.5%), while at 400 °C they were 11 100 and 11 300 mmol g_Ru_^−1^ h^−1^ (+1.7%). The minimal gain from a 60 °C increase between 340 and 400 °C further supports approaching a performance plateau at high thermal input. Testing at 340 °C with increased flow rates (20 and 30 mL min^−1^; WHSV = 30.4 h^−1^ and 45.5 h^−1^) increased the rate from 10 500 to 11 600 mmol g_Ru_^−1^ h^−1^ (+11%), indicating only minor mass transport effects, insufficient to explain the pronounced drop in light‐induced promotion at elevated temperatures.

To examine reaction kinetics, Arrhenius plots of ln *r* versus 1000/*T* were constructed for both conditions (Figure 3d,e) using data from Table S3. Both curves exhibit pronounced curvature, precluding assignment of a single apparent activation energy (*E*_a,app_). Such nonlinearity is common in surface‐catalyzed reactions and can result from temperature‐dependent surface coverages, shifts in the rate‐determining step, or emerging transport effects.^[^ ^41^, ^42^ ^]^ To compare temperature dependence quantitatively, *E*_a,app_ values were extracted as a function of temperature by numerically differentiating the fitted Arrhenius curves according to:

(1) $$r=A\cdot \exp\left(-\frac{E_{a,\mathrm{app}}}{RT}\right)$$

(2) $$\;\ln\left(r\right)=\ln\left(A\right)\;\cdot \frac{E_{a,\mathrm{app}}}{R}\cdot \frac{1}{T}$$

(3) $$E_{a,\mathrm{app}}\;\left(T\right)=-R\cdot \frac{dln\left(r\right)}{d\left(\frac{1}{T}\right)}$$

where *r* is the reaction rate, *A* is the pre‐exponential factor, *E*_a,app_ is the apparent activation energy, *R* is a constant, and *T* is the temperature.

The temperature‐dependent *E*_a,app_ profiles (Figure 3f, Table S4) decrease steadily across the measured range, from 88.6 to 0.3 kJ mol^−1^ in the dark and from 89.7 to −3.9 kJ mol^−1^ under illumination. The close similarity between the two datasets indicates that illumination does not significantly affect the apparent activation barrier, implying that the fundamental reaction pathway remains unchanged. This observation argues against a dominant role of hot‐carrier–driven mechanisms, which would typically produce a marked shift in *E*_a,app_ through the involvement of electronically or vibrationally excited intermediates or other nonthermal activation routes.^[^ ^43^, ^44^, ^45^ ^]^ It should be emphasized that the apparent activation energies derived from kinetic experiments describe the overall macroscopic barrier of the methanation process under steady‐state conditions. The barrier reduction discussed later in the DFT analysis instead reflects the intrinsic electronic stabilization of CO_2_ hydrogenation intermediates at Ru–TiO_2_ interfacial sites induced by metal–support interactions. Thus, illumination does not thermodynamically lower the apparent reaction barrier but accelerates the same Ru‐catalyzed pathway by providing additional plasmonic photothermal energy to these optimized interfacial sites.

Methane was the overwhelmingly dominant product under all conditions. In the dark, CH_4_ selectivity was effectively complete across 170–340 °C, with only a single instance of trace CO formation. Under illumination, a slight but reproducible decrease in CH_4_ selectivity appeared at high temperatures, beginning near 320 °C and reaching ∼1% CO at 340 °C. This minor shift is consistent with localized photothermal heating at the catalyst surface, which can raise the reaction temperature and thermodynamically favor CO formation via the reverse water–gas shift pathway.^[^ ^46^ ^]^ Control experiments at 400 °C in the dark produced similar low CO levels, supporting this interpretation. Overall, the illumination‐induced selectivity change is negligible, confirming that plasmonic excitation primarily accelerates the existing methanation pathway without altering its selectivity profile. While the overall catalytic trends are highly reproducible, some variability in absolute rates was observed between different catalyst batches and upon re‐packing of the same sample. This variability is attributed to subtle differences in Au@Ru nanoparticle deposition onto the TiO_2_ support during catalyst preparation, as well as packing density within the small reactor volume. Importantly, these variations do not affect the key conclusions: (i) intermediate Ru coverages maximize thermal activity, (ii) thin, discontinuous shells preserve strong light responses, and (iii) illumination consistently accelerates methanation without altering selectivity. These observations highlight both the robustness of the structure–activity trends and the need for improved control over nanoparticle deposition to further minimize batch‐to‐batch variability in future studies.

The nearly identical apparent activation energies obtained under dark and illuminated conditions (Figure 3d,f, Table S4) confirm that illumination accelerates the same Ru‐catalyzed methanation pathway rather than altering the underlying mechanism. This observation rules out a hot‐carrier‐dominated process, which would typically produce a measurable shift in activation barrier. Illumination, therefore, enhances turnover along the existing pathway by supplying additional energy through localized plasmonic photothermal heating, with any interfacial electronic effects expected to play only a minor role. This interpretation is consistent with recent quantitative analyses of plasmon‐induced catalysis.^[^ ^47^, ^48^, ^49^ ^]^ The following section explores how this acceleration scales with light intensity and wavelength, providing further evidence that plasmon excitation, rather than a change in reaction route, is responsible for the observed rate enhancement.

**Figure 4:**
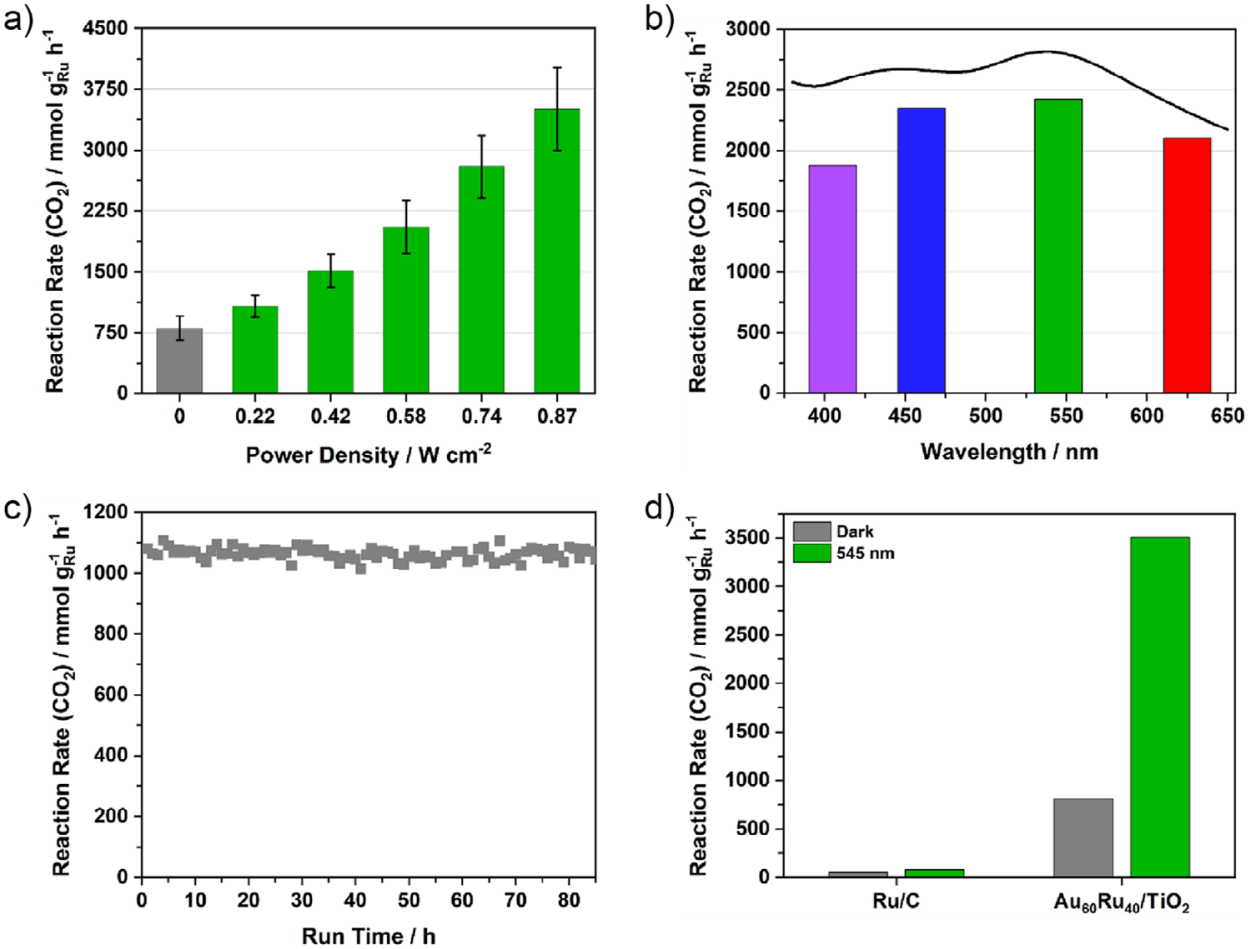
Light‐intensity, wavelength, stability, and benchmarking studies for Au_60_Ru_40_/TiO_2_. a) CO_2_ methanation rate as a function of 545 nm power density at 190 °C, showing a super‐linear enhancement consistent with photothermal promotion. b) Action spectrum at equal power density (0.67 W cm^−2^) from 400–650 nm, peaking near the LSPR maximum (545 nm) and tracking the optical absorption profile. c) Long‐term stability test at 190 °C (dark), showing stable performance over 85 h. d) Benchmarking against commercial 5 wt % Ru/C under identical conditions (190 °C, dark and illuminated), demonstrating that Au_60_Ru_40_/TiO_2_ delivers higher thermal activity and a much stronger light response than the non‐plasmonic reference.

The effect of incident light intensity on CO_2_ methanation using the Au_60_Ru_40_/TiO_2_ catalyst was examined at 190 °C under 545 nm illumination, matching the LSPR of the supported nanoparticles (Figure 4a; relative rate increases are depicted in Figure S6). In the dark, Au_60_Ru_40_/TiO_2_ achieved 806 mmol g_Ru_^−1^ h^−1^. Even at the lowest tested intensity (0.22 W cm^−2^), the rate increased by ∼33% to 1100 mmol g_Ru_^−1^ h^−1^. Higher power densities produced progressively larger enhancements, reaching 3500 mmol g_Ru_^−1^ h^−1^ at 0.87 W cm^−2^ (∼335% enhancement). The enhancement did not scale linearly with light intensity but followed an exponential‐like trend at higher intensities (0.58–0.87 W cm^−2^), consistent with a dominant photothermal contribution in which the local temperature rise scales linearly with photon flux while the reaction rate increases exponentially via the Arrhenius relationship.^[^ ^8^, ^43^, ^50^, ^51^ ^]^ Although hot‐carrier effects may contribute, particularly at lower intensities, the present data cannot unambiguously separate them from photothermal effects. Overall, the results reveal a strong, non‐linear positive correlation between visible‐light intensity and methanation activity.

Wavelength‐dependent activity was evaluated at 190 °C under equal power density (0.67 W cm^−^^2^) across 400–650 nm (Figures 4b and Figure S7). In the dark, the rate was 723 mmol g_Ru_^−1^ h^−1^. Illumination at 545 nm, coinciding with the LSPR maximum, yielded the highest rate (2400 mmol g_Ru_^−1^ h^−1^), consistent with plasmonic excitation of the Au core as the dominant promotion pathway. Excitation at 460 nm gave a comparable rate (2350 mmol g_Ru_^−1^ h^−1^), reflecting strong absorbance near but slightly blue‐shifted from the LSPR peak. Even at 625 nm, where absorbance is reduced, a substantial enhancement was maintained (2110 mmol g_Ru_^−1^ h^−1^), indicating the broad spectral width of the plasmon resonance and possible additional absorption by the Ru shell at longer wavelengths.^[^ ^8^ ^]^ At 405 nm, the rate (1876 mmol g_Ru_^−1^ h^−1^) was noticeably lower despite significant optical absorbance in the UV–vis spectrum. This decrease is attributed to interband transitions in Au, which dominate above ∼2.4 eV (∼520 nm).^[^ ^52^, ^53^ ^]^ Although optically intense, these transitions are less effective at generating plasmonic hot carriers or localized photothermal heating for methanation, explaining the lower activity relative to the resonant excitation.^[^ ^8^ ^]^ Across all wavelengths, CH_4_ selectivity remained above 99%, indicating that illumination primarily affects the reaction rate rather than the reaction pathway. The alignment between the activity maximum and the LSPR peak underscores the central role of Au‐core plasmonic excitation in driving methanation, either via localized photothermal heating or hot‐carrier generation at the Au‐Ru interface.

The light‐intensity and wavelength‐dependent results confirm that plasmonic excitation of the Au core is the primary driver of the observed activity enhancements in Au_60_Ru_40_/TiO_2_. The pronounced wavelength dependence (Figure 4b and Figure S7), peaking near the Au plasmon resonance, together with the nearly identical apparent activation energies under dark and illuminated conditions (Figure 3d–f, Table S3), shows that illumination accelerates the same Ru‐catalyzed methanation pathway rather than inducing a new, nonthermal mechanism. While Au can generate both hot carriers and localized heating, distinguishing their respective roles and whether hot carrier excitation of intermediates occurs at Au or Ru sites is inherently challenging. Control experiments using Au/TiO_2_ and Ru/TiO_2_ under identical illuminated conditions (Figure S8) yielded negligible methanation activity, confirming that direct CO_2_ activation on isolated Au or Ru sites is insignificant. Instead, the enhancement can be attributed to plasmonic photothermal heating, with possible minor interfacial hot‐carrier contributions, that promote turnover at Ru and Ru–TiO_2_ interfacial sites. The thin, discontinuous Ru shell in Au_60_Ru_40_/TiO_2_ preserves strong Au plasmon absorption while providing abundant accessible Ru active sites, enabling efficient light‐to‐heat coupling at the interface. In contrast, thicker Ru shells dampen the plasmonic resonance, limiting light harvesting despite higher intrinsic thermal activity. The minimal changes in CH_4_ selectivity, with only trace CO formation at elevated temperatures under illumination, further support a mechanism dominated by accelerated methanation via photothermal promotion rather than pathway switching. These findings highlight the importance of balancing plasmonic accessibility and catalytic site density in the design of optically active core–shell catalysts for light‐assisted thermocatalysis^[^ ^24^, ^25^ ^]^ and naturally connect to the DFT analysis discussed later in the manuscript. In combination with the commercial Ru/C benchmark (Figure 4d) and the wavelength‐ and intensity‐dependent trends (Figures 4a,b, S6, and S7). These results provide a comprehensive dataset supporting the plasmonic origin of the observed promotion.

The thermal stability of Au_60_Ru_40_/TiO_2_ was assessed during continuous CO_2_ methanation at 190 °C without illumination for 85 h (Figure 4c). The CH_4_ formation rate remained steady (average 1070 mmol g_Ru_^−1^ h^−1^) with no detectable deactivation or induction period; minor fluctuations were within experimental uncertainty. Such durability is essential for continuous‐flow implementation of plasmonic‐promoted catalysts. Post‐reaction TEM analysis of Au_60_Ru_40_/TiO_2_ after 85 h of continuous operation (Figure S9) revealed no detectable sintering, aggregation, or carbon deposition. The nanoparticles retain the same morphology and size distribution as prior to reaction (Figure S4), confirming that the Au@Ru core–shell structure remains structurally intact under reaction conditions. This structural stability is consistent with the excellent catalytic durability and constant CH_4_ selectivity observed during extended operation (Figure 4c).

Benchmarking against a commercial 5 wt % Ru/C reference under identical conditions (190 °C, dark and 545 nm at 0.87 W cm^−2^; Figure 4d) further underscored the advantage of the core–shell design. In the dark, Ru/C achieved 54 mmol g_Ru_^−1^h^−1^, rising to 81 mmol g_Ru_^−1^ h^−1^ under illumination (∼49% enhancement from C and Ru photothermal effects), consistent with its previously reported behavior as a purely photothermal catalyst in CO_2_ methanation.^[^ ^54^ ^]^ In contrast, Au_60_Ru_40_/TiO_2_ delivered 806 mmol g_Ru_^−1^ h^−1^ in the dark and 3510 mmol g_Ru_^−1^ h^−1^ under illumination (∼335% enhancement), combining nearly threefold higher thermal activity with a far greater light response than the non‐plasmonic benchmark.

While in situ spectroscopic studies could, in principle, provide complementary information, such measurements are experimentally challenging under the high‐temperature, flowing‐gas, and illuminated conditions used here; therefore, mechanistic interpretation was instead derived from steady‐state kinetics and DFT calculations, which together capture the key surface and interfacial processes.

**Figure 5:**
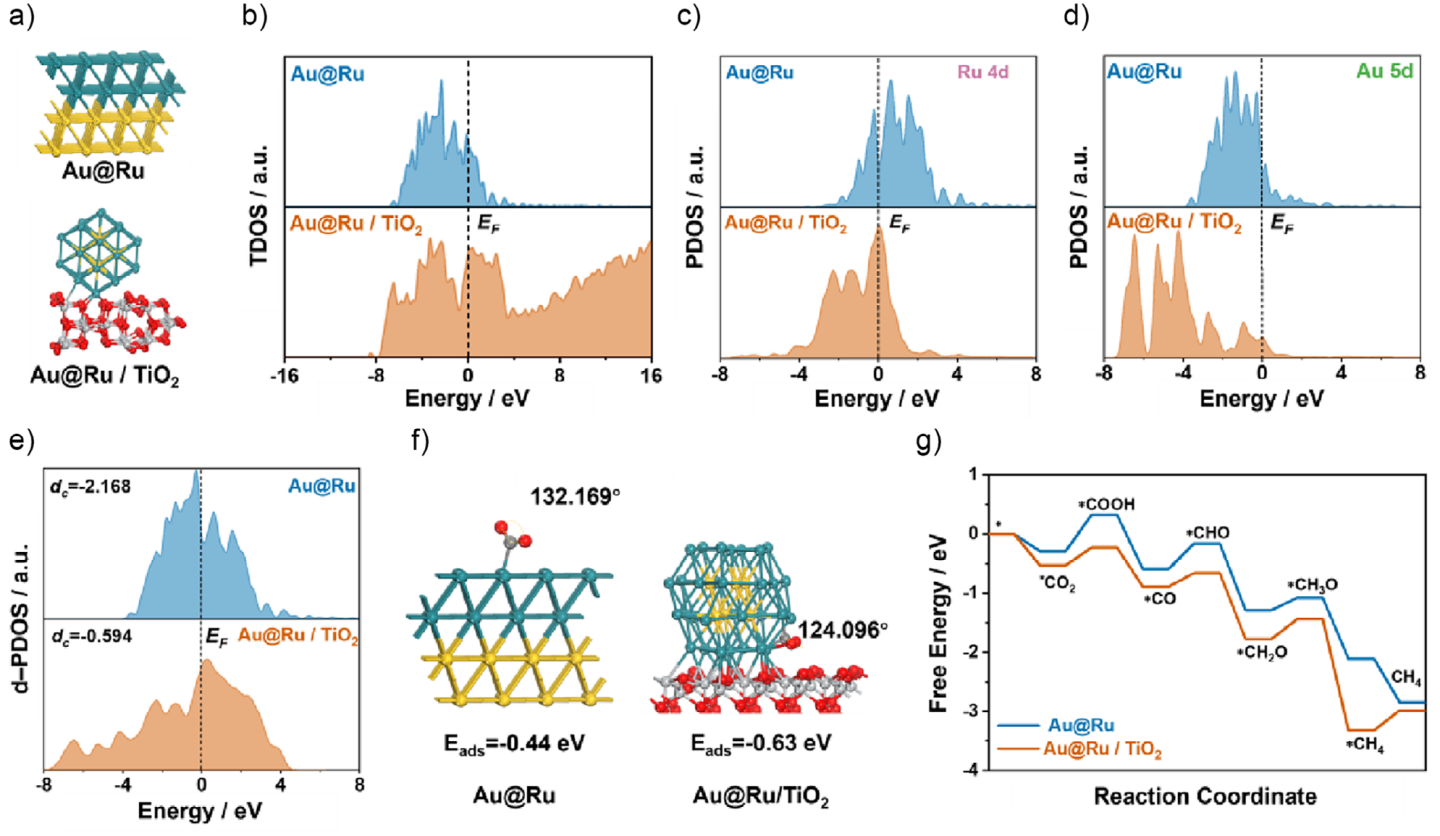
DFT insights into electronic structure, CO_2_ activation, and reaction pathways. a) Models of unsupported Au@Ru(111) and Au@Ru/TiO_2_(110) surfaces. b) Total DOS showing a broader unoccupied state distribution for Au@Ru/TiO_2_, enabling greater acceptor‐state availability for hot electrons. c, d) PDOS for Ru 4d and Au 5d orbitals showing downshifts upon TiO_2_ support, indicative of modified interfacial coupling. e) d‐band center analysis showing Au@Ru/TiO_2_ shifted closer to the Fermi level than Au@Ru, favoring weaker binding of strongly adsorbed intermediates. f) Optimized CO_2_ adsorption geometries showing stronger binding and greater bending at the Au@Ru/TiO_2_ interface than on unsupported Au@Ru. g) Free‐energy profiles for CO_2_ methanation showing a lower barrier for the first hydrogenation step and a shift in the rate‐limiting step from *CO_2_ → *COOH (unsupported) to *CH_4_ desorption (supported). These results confirm that TiO_2_ induces strong metal–support interactions, optimizes Ru electronic structure, and reduces key kinetic barriers, in agreement with experimental observations.

To gain atomistic insight into the enhanced CO_2_ methanation activity of Au@Ru/TiO_2_, density functional theory (DFT) calculations were performed to probe the electronic structure, CO_2_ activation, and the complete reaction pathway. Two slab models were examined: a pristine Au@Ru(111) surface and Au@Ru supported on TiO_2_(110) (Figure 5a). Charge density difference maps (Figure S10) reveal substantial electron redistribution at the Au@Ru/TiO_2_ interface, with electron accumulation on Ru and depletion on TiO_2_ surface oxygen, hallmarks of strong metal–support interaction.^[^ ^55^ ^]^ Milliken charge analysis confirms more efficient electron migration in the supported system, which should facilitate both CO_2_ activation and photo‐induced charge transfer under illumination.

Electronic structure analysis supports this picture. The total density of states (TDOS) for Au@Ru/TiO_2_ (Figure 5b) shows an extended distribution of unoccupied states above the Fermi level compared to Au@Ru, providing a larger reservoir of acceptor states accessible thermally or via hot‐electron injection. Projected DOS (PDOS) for the Ru 4d and Au 5d orbitals (Figures 5c,d, S11, and S12) show downshifts in both bands for the supported catalyst, indicative of modified electronic coupling at the interface.^[^ ^56^ ^]^ d‐band center analysis (Figure 5e) reveals that Au@Ru/TiO_2_ has a d‐band center (−0.594 eV) closer to the Fermi level than Au@Ru, which can weaken the binding of strongly adsorbed intermediates and improve turnover.^[^ ^57^ ^]^ These results point to an electronic environment in Au@Ru/TiO_2_ being better tuned for the catalytic activation than in the unsupported nanoparticle.

CO_2_ adsorption calculations further illustrate this effect. On Au@Ru(111), CO_2_ binds with an energy of − 0.44 eV, bending from 180° in the gas phase to 132° upon adsorption (Figure 5f). At the Au@Ru/TiO_2_ interface, binding is stronger (−0.63 eV) and the molecule is more distorted (124°), indicating deeper activation and greater susceptibility to C─O bond cleavage.^[^ ^58^ ^]^ Bare TiO_2_ (110) interacts only weakly with CO_2_, with negligible bond distortion, confirming the metal–support interface as the primary active site.

The full CO_2_ methanation pathway (Figure 5g) shows that initial CO_2_ adsorption is exothermic in both models, but the first hydrogenation to *COOH is more favorable on Au@Ru/TiO_2_ (barrier 0.30 eV) than on Au@Ru (0.61 eV). Subsequent C─O bond cleavage and hydrogenation steps proceed with low barriers, yielding CH_4_ selectively. For Au@Ru/TiO_2_, the rate‐limiting step is *CH_4_ desorption (0.34 eV), whereas for Au@Ru it is the *CO_2_ → *COOH step, with a substantially higher barrier. Thus, the TiO_2_ support lowers the key kinetic barriers and shifts the rate‐limiting step to a later, less energetically demanding stage.

These DFT results provide a molecular‐level rationale for the experimentally observed superiority of Au@Ru/TiO_2_ in both dark and illuminated CO_2_ methanation. The TiO_2_ support induces strong metal–support interactions and tunes the Ru d‐band position to optimize intermediate binding. This enhanced electronic configuration, coupled with the experimentally demonstrated LSPR activity of the Au core, generates a dual‐promotional effect: (i) thermally, by lowering activation barriers for CO_2_ activation and hydrogenation at the interface, and (ii) photothermally, by enabling efficient hot‐carrier or heat‐assisted population of the extended unoccupied states above the Fermi level. The agreement between the reduced reaction barriers in DFT and the enhanced reaction rates under illumination confirms that the same interfacial sites optimized for thermal catalysis are also the most responsive to plasmonic excitation, completing the structure–property–function relationship established in this work. This correlation arises because TiO_2_‐induced metal–support interactions electronically tune the Ru 4d states, strengthening CO_2_ adsorption and lowering the intrinsic barrier for the first hydrogenation step. Under illumination, the localized surface plasmon resonance of the Au core concentrates visible light into near‐field energy that is efficiently delivered as localized heat to these same Ru–TiO_2_ interfacial sites. Consequently, the Ru sites optimized for thermal catalysis are also the most effectively promoted by plasmonic excitation, completing the structure–performance–function relationship in Au@Ru/TiO_2_. It is worth noting that the DFT‐derived decrease in reaction barriers represents intrinsic structural and electronic optimization of the Au–Ru–TiO_2_ interface rather than a light‐induced modification of the experimental activation energy.

## Conclusion

We have demonstrated that Au@Ru core–shell nanoparticles with tunable Ru coverage, when supported on TiO_2_, serve as highly active and light‐responsive catalysts for CO_2_ methanation. Systematic variation of shell thickness revealed that the Au_60_Ru_40_ composition, with a thin, discontinuous Ru shell, achieves the optimal balance between Ru active‐site density and preservation of the Au core's LSPR. Under visible‐light excitation, this catalyst exhibited up to a 335% activity enhancement over dark conditions, far exceeding the performance of a commercial Ru/C benchmark, while maintaining exceptional thermal stability over 85 h of continuous operation. Correlative structural (TEM, HAADF‐STEM, and XPS), optical (UV–vis), and catalytic measurements established a direct structure–performance relationship: thin Ru shells maximize plasmonic light harvesting without sacrificing access to hydrogenation sites. Kinetic analysis showed that illumination accelerates the methanation pathway without altering the rate‐determining step, consistent with a plasmonic photothermal mechanism. DFT calculations confirmed that the TiO_2_ support induces strong metal–support interactions, shifts the Ru d‐band center, strengthens CO_2_ adsorption, and lowers the barrier for the first hydrogenation step, shifting the rate‐limiting step to CH_4_ desorption. These combined experimental and theoretical insights reveal a dual‐promotional effect that explains the large performance enhancement observed under illumination. Thermally, metal–support interactions at the Ru–TiO_2_ interface optimize the electronic structure for CO_2_ activation and hydrogenation, lowering intrinsic reaction barriers. Photothermally, excitation of the Au core's localized surface plasmon resonance concentrates visible light into localized fields that are converted into heat, and possibly minor interfacial electronic excitation, thereby accelerating turnover at these same Ru–TiO_2_ sites without altering the fundamental reaction pathway. The wavelength‐ and intensity‐dependent trends, together with DFT analysis, confirm that illumination amplifies the activity of already‐optimized interfacial sites, establishing a clear structure–performance–function relationship in Au@Ru/TiO_2_. Beyond CO_2_ methanation, this work highlights nanoscale shell engineering as a powerful and generalizable strategy for designing high‐efficiency plasmonic catalysts across a broad range of light‐assisted, energy‐relevant transformations.

## Conflict of Interests

The authors declare no conflict of interest.

## Supporting information



Supporting Information

## Data Availability

The data that support the findings of this study are available from the corresponding author upon reasonable request.
