# Supplementary material for: Plasmon‐Enhanced CO2 Methanation over Au@Ru/TiO2 via Nanoscale Control of Ru Shell Thickness
Source: Angew Chem Int Ed Engl. 2025 Nov 3;64(52):e18748. doi: 10.1002/anie.202518748 (PMC12723468; doi:10.1002/anie.202518748)
Supplement: Supplementary file 1 — Supporting Information [file ANIE-64-e18748-s001.pdf]

Supporting Information for

Plasmon-Enhanced CO_2 Methanation over Au@Ru/TiO_2 via Nanoscale Control of Ru Shell Thickness

Florian Rathmann,[1,2†] IbrahiM Abdelsalam,[2†] Shiqi Wang,[2] Maja M. Kubik,[2] Sana Frindy,[2] Tiago V. Alves,[3] Mykhailo Chundak,[2] Mikko Ritala,[2] Alexander Reznichenko,[1] Matti Reinikainen,[1] and Pedro H. C. Camargo,[2,*]

[1] *VTT Technical Research Centre of Finland, P O Box 1000, FIN-02044 Espoo, Finland*

[2] *Department of Chemistry, University of Helsinki, A.I. Virtasen aukio 1, PO Box 55, FIN-0014 Helsinki, Finland*

[3] *Departamento de Físico-Química, Instituto de Química, Universidade Federal da Bahia, Rua Barão de Jeremoabo, 147, Salvador, BA, 40170-115, Brazil*

**Corresponding author. Email: pedro.camargo@helsinki.fi*

[†]These two authors contributed equally to this work.

Experimental Section

Materials and Instrumentation

Hydrochloroauric acid trihydrate ($HAuCl_4 \cdot 3H_2O$), ruthenium(III) chloride hydrate ($RuCl_3 \cdot 3H_2O$), trisodium citrate ($Na_3C_6H_5O_7$), and titanium dioxide (TiO_2 nanopowder, 21 nm particle size, CAS 13463-67-7) were purchased from Sigma-Aldrich and used as received without further purification. All glassware was rigorously cleaned with freshly prepared aqua regia ($HCl:HNO_3$ = 3:1) and rinsed thoroughly with ultra-pure water (resistivity = 18.2 MΩ·cm) prior to use. Ultra-pure water was also used for all synthesis steps and washing procedures.

Scanning electron microscopy (SEM) was performed using a Hitachi S-4800 field-emission SEM operated at 10 kV with a working distance of 8.5 mm. Samples for SEM analysis were prepared by centrifugation and washing with water, followed by drop-casting the nanoparticle dispersion onto pieces of silicon wafers. Transmission electron microscopy (TEM) was conducted on a JEOL JEM-1400 microscope. For TEM, samples were prepared by dispersing the nanoparticles in ethanol using an ultrasonic bath, followed by drop-casting onto carbon-coated copper grids. High-resolution TEM and EDS mapping were performed using JEOL JEM-2200FS. Optical absorption spectra of the nanoparticle dispersions were recorded using a Shimadzu UV-2600 spectrophotometer in the 300–850 nm range with a step size of 0.5 nm.

Powder X-ray diffraction (PXRD) data were collected on a Bruker D8 Advance in Bragg−Brentano geometry using Cu Kα radiation (λ = 1.5406 Å) with a Ni filter. Diffraction data were collected over a range of 10−80° 2θ (step width 0.02° 2θ, count time 1 s/step). The diffraction patterns have been indexed by comparison with the Joint Committee on Powder Diffraction Standards (JCPDS) files. The elemental composition analysis was performed by Microwave Plasma Atomic Emission Spectroscopy (MP-AES) using an Agilent Technologies 4100 MP AES. Three independent measurements were performed for each sample. The samples were prepared by digesting the

Au@Ru nanoparticles in 7 mL of an acid mixture ($HCl:HNO_3$ = 6:1). The resulting solution was placed in High-Density Polyethylen (HDPE) vessels and heated at 90 °C for 3 hours. After cooling the vessels to room temperature, the samples were diluted to a final volume of 50 mL with water. A 10 mL aliquot of each solution was then analyzed by MP-AES. Calibration standards for Au and

Ru were prepared from $HAuCl_4 \cdot 3H_2O$ and $RuCl_3 \cdot 3H_2O$ standard solutions, respectively, both dissolved in HCl (purchased from Sigma-Aldrich)

X-ray photoelectron spectroscopy (XPS) spectra were acquired using a PREVAC spectrometer with a monochromatized Al Kα anode (1486.7 eV) under ultrahigh vacuum (10^{-10} mbar). Data interpretation was performed via Casa XPS. A Shirley or two-point linear background was used depending on the spectrum shape.

Surface site accessibility and CO_2 interaction strength were evaluated through pulsed H_2 chemisorption, static CO_2 chemisorption, and CO_2 temperature-programmed desorption (TPD), using a Micromeritics 3Flex surface characterization analyzer equipped with a thermal conductivity detector (TCD) maintained at 175 °C. All experiments were performed sequentially on a single $Au_{60}Ru_{40}/TiO_2$ catalyst sample (0.642 g). The sample was first reduced in pure H_2 (10 mL min^{-1}) by heating to 350 °C at a ramp rate of 15 °C min^{-1}, maintaining this temperature for 90 min, and subsequently cooling to ambient temperature. After cooling, the gas flow was switched to Ar (50 mL min^{-1}) and the sample was reheated to 350 °C with a ramp rate of 15 °C min^{-1} for 60 min to remove residual hydrogen. Pulsed H_2 chemisorption was performed using a pulse loop volume of 0.4921 mL at a loop temperature of 45 °C, with a 10% H_2/Ar mixture. Following H_2 chemisorption, the sample was purged under Ar at 350 °C for 90 min and cooled to ambient. Static CO_2 chemisorption was subsequently performed at 35 °C after evacuation and surface equilibration. Following this, CO_2 TPD was carried out under He flow (10 mL min^{-1}) after saturation with CO_2 pulses (0.4921 mL). The temperature was ramped to 900 °C at 10 °C min^{-1} and held for 10 min at the final temperature.

Synthesis of Au@Ru Nanoparticles

Monometallic Au nanoparticles (NPs) were synthesized by citrate reduction of $HAuCl_4$.[1] In a typical synthesis, 100 mL of a 0.25 mM $HAuCl_4$ aqueous solution was heated to boiling in a 250 mL round-bottom flask. Upon boiling, 3 mL of 1 wt % trisodium citrate was added, and the solution was refluxed for 10 min, during which the color changed to deep red, indicating formation of Au NPs. The dispersion was allowed to cool to room temperature.

Bimetallic Au@Ru nanoparticles were prepared by a self-catalyzed deposition of Ru onto preformed Au seeds without additional reducing agents.[2] In a typical procedure, 41.3 mL of the Au NP solution (2.42×10^{-4} M) was transferred to a 100 mL round-bottom flask. Varied volumes of 5.2 mg mL^{-1} $RuCl_3$ solution (0.2, 0.4, 0.8, and 2 mL) were added to obtain different Au:Ru molar ratios (1:0.4, 1:0.8, 1:1.6, and 1:4, respectively). The mixture was heated to 70 °C and maintained under stirring for 2 h. As Ru deposition proceeded, the solution color changed from red to violet (low Ru) or brown (high Ru).The samples were named according to their atomic ratio as shown in **Table S1**.

Preparation of Au@Ru NPs supported on TiO_2 (Au@Ru/TiO_2)

To prepare the supported catalyst as in case of $Au_{60}Ru_{40}$, 70 mg of TiO_2 was added to the whole aqueous Au@Ru NPs suspension (Au $2.4 \cdot 10^{-4}$ M). The pH was adjusted to 3 by adding 20 µL of concentrated HNO_3. The suspension was refluxed at 70 °C overnight. The solid product was collected via centrifugation and washed multiple times with water. The recovered catalyst was dried at 60 °C for 24 h, gently ground with a mortar and pestle, and stored in a glass vial for further use. The same procedures were used for other compositions without any modifications.

Photoreactor Configuration and Irradiation Setup

Photocatalytic experiments were conducted in a custom-built setup using a Harrick High Temperature Reaction Chamber (HVC-MRA-5) equipped with a quartz window for light access. Temperature was precisely controlled using a Harrick ATC-024-3 controller with the thermocouple positioned directly within the catalyst bed to ensure accurate bulk temperature monitoring. This configuration enabled stable operation under both dark and illuminated conditions, minimizing any confounding effects from photothermal heating. A schematic of the reactor and a photograph under operating conditions are shown in **Figure S1.** Light irradiation was provided by a Multi-Wavelength Fiberoptic LED Light Source (Prizmatix), allowing selection between 405, 460, 545, and 625 nm wavelengths. The corresponding spectral profiles are shown

in **Figure S2**. Irradiance was calibrated prior to each experiment using a Thorlabs PM400 power meter with an S425C sensor to ensure consistent light intensity across all wavelengths.

Catalytic Tests

All catalytic experiments were conducted using 20 mg of the respective supported Au@Ru catalysts. Prior to testing, each catalyst was reduced in situ at 350 °C for 1 h under a continuous flow of H_2/Ar (10 vol %, ± 2% relative uncertainty) at 50 mL min^{-1}, ramping at 15 °C min^{-1}. The reaction gas mixture consisted of H_2, CO_2, and N_2 in a volumetric ratio of 72:18:10 (± 2% relative uncertainty for each component), supplied by Linde Gas AB. Flow rates of the reducing and reaction gas mixtures were regulated using a Bronkhorst mass flow controller (F-201D-RAA-33-E), calibrated under standard conditions. Reactions were conducted at ambient pressure under both dark and illuminated conditions.

During catalytic testing, the catalyst-bed temperature was continuously monitored using a thermocouple embedded directly within the packed bed to ensure accurate bulk temperature measurement (**Figure S1**). For each data point, the system was allowed to equilibrate for at least 20 minutes under the target temperature and illumination conditions before gas sampling. Reaction products were analyzed only after the temperature had fully stabilized at the externally set level, ensuring that all reported rates reflect steady-state operation rather than transient light-induced temperature fluctuations. The near-identical apparent activation energies obtained under dark and illuminated conditions (**Figures 3D–F**, **Table S4**) further confirm that the measurements represent true steady-state behavior, free from thermal artifacts during light switching.

Temperature-dependent studies were carried out between 170 and 340 °C, in 10 °C increments, conducted under both dark conditions and continuous 545 nm illumination at a constant power density of 0.67 W cm^{-2} For these measurements, the total gas flow rate was set to 20 mL min^{-1} (eight Hourly Space Velocity, WHSV = 30.4 h^{-1}) to avoid potential mass transfer limitations at higher temperatures. Each experiment was performed at least two times to ensure repeatability. All other catalytic tests were conducted at a total flow rate of 11.8 mL min^{-1} (WHSV = 17.8 h^{-1}).

The influence of photon flux on the reaction rate was evaluated by varying the incident power density of the 545 nm LED light between 0.22 and 0.87 W cm^{-2} at a fixed reaction temperature of 190 °C. In addition, wavelength-dependent measurements were conducted at 405, 460, 545, and 625 nm, each applied at a fixed power density of 0.67 W cm^{-2} and a constant temperature of 190 °C. To ensure reliability, light intensity-dependent experiments were repeated four times and wavelength-dependent experiments five times.

Catalytic activity is expressed as the reaction rate in millimoles of CO_2 converted per gram of Ru per hour (mmol g_{Ru}^{-1} h^{-1}). At 190 °C, under both dark and illuminated conditions, CH_4 was the main carbonaceous product observed, with only trace amounts of ethane and propane detected (<0.01 vol. %), which were excluded from the selectivity analysis due to their low concentrations. Thus, all reaction rate figures are plotted without selectivity axis. Reaction products and CO_2 conversion were analyzed via gas chromatography (Agilent 6890N), employing a thermal conductivity detector(TCD) with a packed Carboxen-1000 column for CO_2 and CO quantification, and a flame ionization detector (FID) coupled to a capillary Al_2O_3 column for hydrocarbon detection. Calibration of both detectors was performed using certified reference gases. Control experiments with the blank reactor, conducted without catalyst at 190 °C under dark conditions and under 545 nm irradiation at 0.87 W cm^{-2}, confirmed the lack of conversion without catalyst. Catalyst stability was evaluated by a long-term test under dark conditions at 190 °C for 86 hours using the $Au_{60}Ru_{40}/TiO_2$ catalyst. A commercial Ru/C catalyst (5 wt %, Sigma-Aldrich) was tested as a benchmark under identical reaction conditions (190 °C, 11.75 mL min^{-1}, dark and 545 nm illumination at 0.87 W cm^{-2}). While all catalytic tests were repeated to confirm consistency, some variability in absolute rates was observed between different catalyst batches and upon re-packing of the same sample. These differences likely arise from subtle variations in catalyst deposition over the support and packing density within the small reactor volume.

Theoretical calculations

DFT calculations were performed with the first-principles simulation Cambridge Sequential Total Energy Package (CASTEP) module in Materials Studio software.[3] The exchange-correlation potential was described by the generalized gradient approximation (GGA) with the Perdewe-Burkee-Ernzerhof (PBE) functional.[4] Interactions between valence electrons and ionic cores

were described by the OTFG ultrasoft pseudo-potential method. A plane-wave basis set with a cutoff energy of 380 eV was assigned to the potential method. The empirical dispersion correction in Grimme's scheme was employed to consider the van der Waals (vdW) interaction. The Broyden-Fletcher-Goldfarb-Shannon (BFGS) algorithm with a medium quality setting of k-points was used for all the energy minimizations in this work. The geometry optimization convergence tolerances for the energy change, maximum force and maximum displacement were 5×10^{-5} eV/atom, 0.001 eV/Å, and 0.005 Å, respectively. For all the models, a 20 Å vacuum space was set in the z-axis to guarantee full relaxation.

The discrete dipole approximation (DDA) was employed to calculate the electric field enhancement contours $|E|^2/|E_0|^2$ for Au nanoparticle cores 15 nm in diameter. Based on the experimental evidence, the Ru thickness in Au nanospheres was kept at 2, 4, and 7 nm. The local electric field distribution was calculated considering a plane located 2 nm away from the metallic surface. The excitation wavelength used in the near-field calculations was 545 nm, and the polarization direction for the sphere and the shell was along the y-axis. For the cubic grid, we employed a spacing of 0.5 nm for all nanostructures. The medium refractive index was 1.0 (air) in all cases. The near fields were described by a grid of 4.0 x 104 points on the yz plane, the dielectric constants for Au and Ru were obtained from the literature.[5, 6] The DDA Convert Tool[7] was used to convert a non-standard geometry (Ru@Au nanoparticles), generated by Blender 3D editor[8], to a collection of dipoles used by the DDSCAT 7.3 in the resolution of a 3N complex linear equations system as implemented by Draine and collaborators.[9-13]

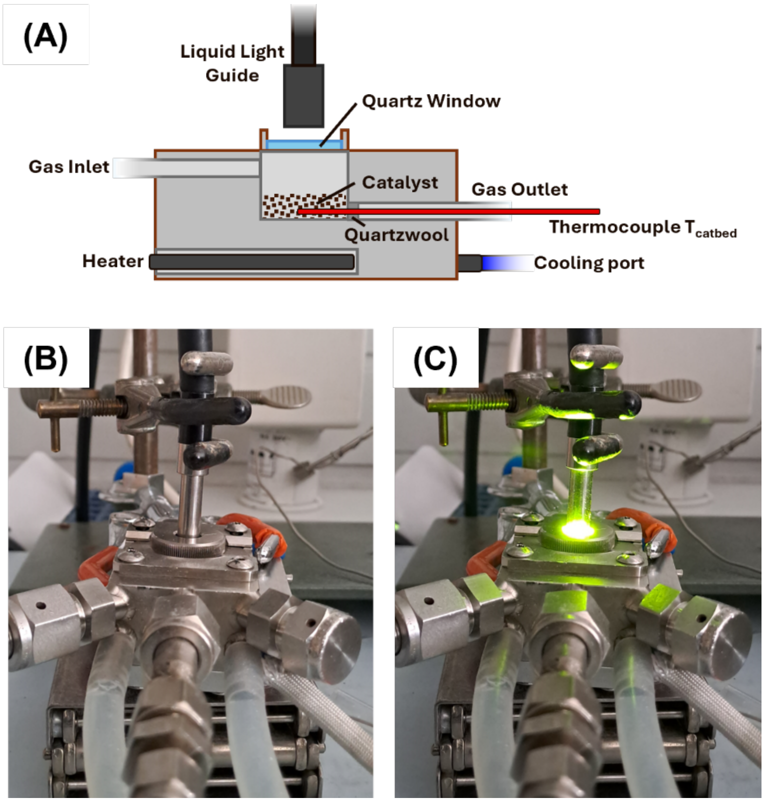

Figure S1. Custom-built photocatalytic reactor. (A) Schematic of the reaction chamber with a quartz window for light access, quartz wool-packed catalyst bed, and thermocouple embedded within the bed for accurate temperature measurement. (B, C) Photographs of the reactor operating under (B) dark and (C) 545 nm LED illumination, showing the experimental configuration for thermal and light-assisted CO_2 methanation studies.

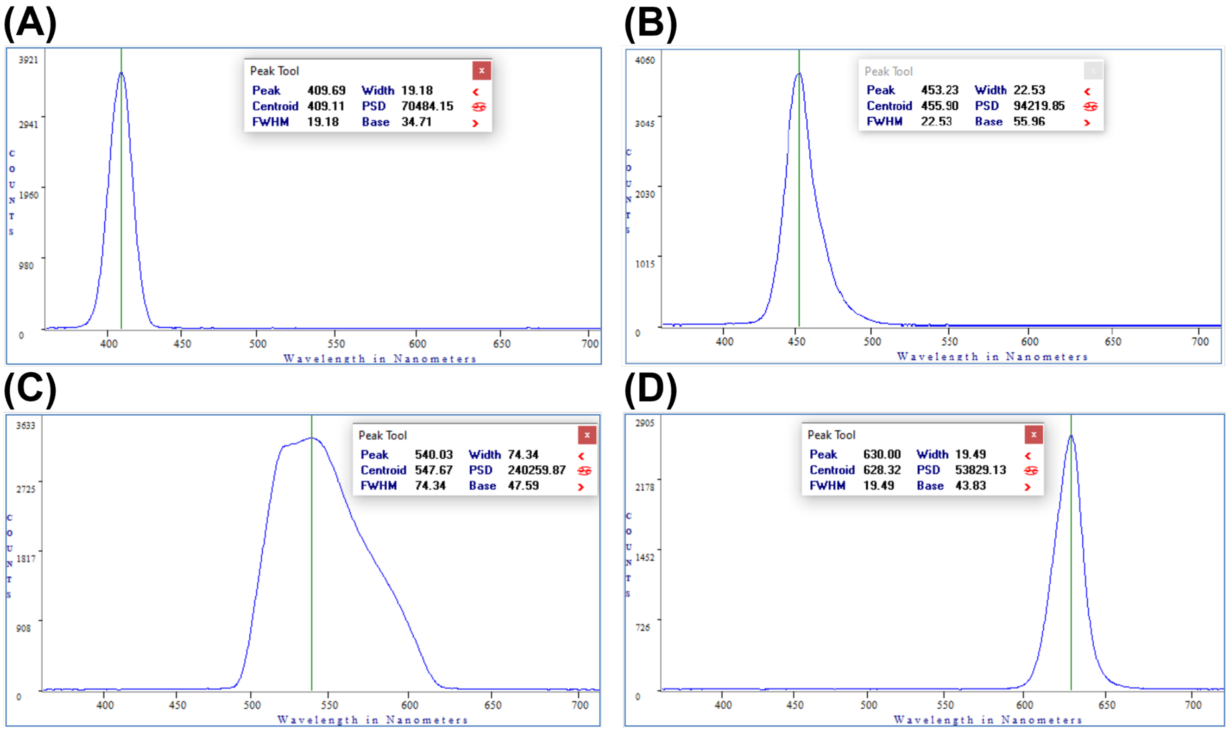

Figure S2. Emission profile of LED light sources. Spectral output of fiber-coupled LEDs used for photocatalytic testing: (A) 405 nm, (B) 460 nm, (C) 545 nm, and (D) 625 nm. Peak wavelength and FWHM are indicated for each source, confirming narrow-band emission suitable for wavelength-dependent studies. Data provided by the manufacturer (Prizmatix).

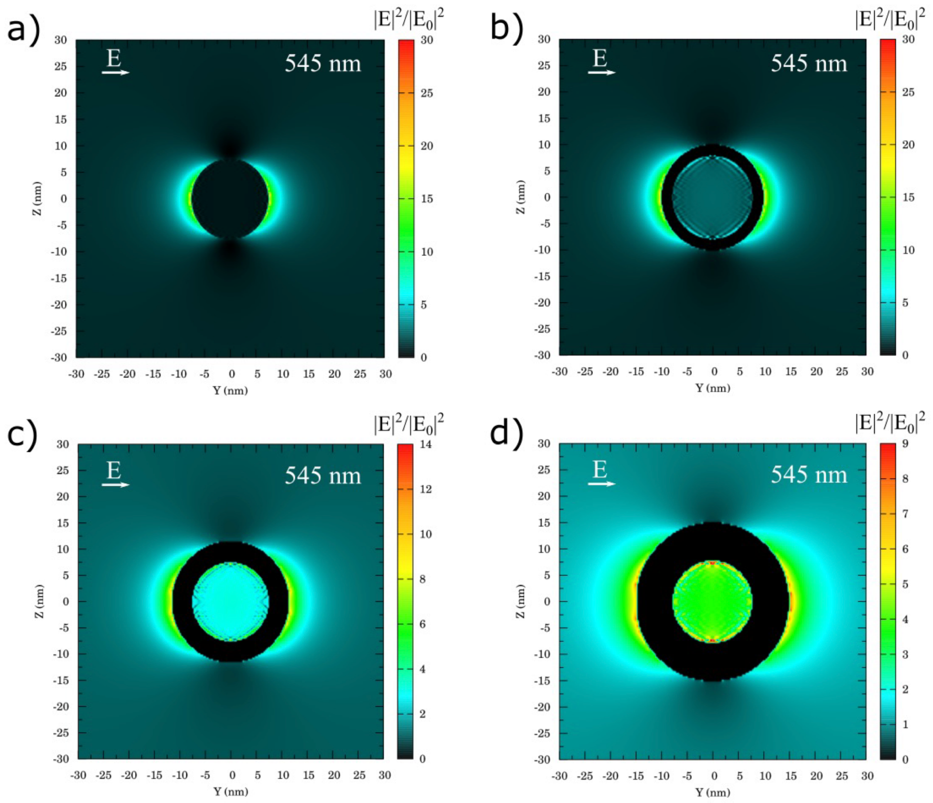

Figure S3. DDA-simulated near-field distributions and field enhancement for Au@Ru nanoparticles. Simulated electric-field intensity maps ($|E|^2/|E_0|^2$) at 545 nm for (a) Au nanoparticles and (b–d) Au@Ru core–shell structures with Ru shell thicknesses of 2, 4, and 7 nm, respectively. The calculations were performed using the discrete dipole approximation (DDA). A progressive damping of the localized surface plasmon resonance and attenuation of near-field intensity are observed with increasing Ru coverage, reflecting optical screening of the Au core. The maximum field enhancement (E^2_{max}) decreases from 29.5 (Au) to 25.8 (2 nm Ru), 12.1 (4 nm Ru), and 8.2 (7 nm Ru), confirming that thin, discontinuous Ru shells preserve strong plasmonic coupling, whereas thicker shells increasingly suppress the local electromagnetic field.

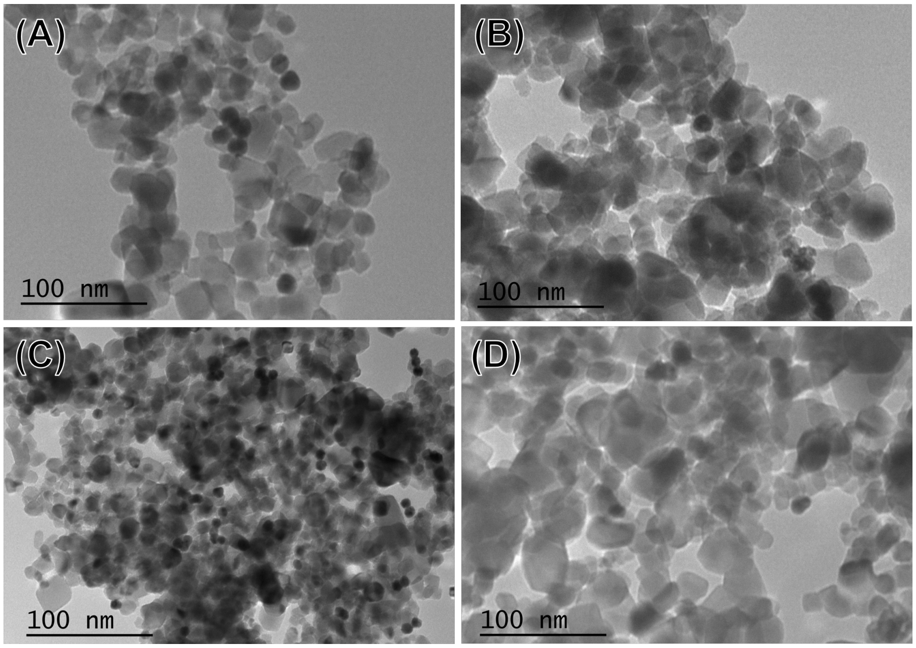

Figure S4. TEM of supported Au@Ru/TiO_2 catalysts. Representative TEM images of (A) $Au_{76}Ru_{24}/TiO_2$, (B) $Au_{60}Ru_{40}/TiO_2$, (C) $Au_{43}Ru_{57}/TiO_2$, and (D) $Au_{22}Ru_{78}/TiO_2$ catalysts, showing the dispersion of Au@Ru NPs on the TiO_2 support with no evidence of aggregation or morphological degradation after immobilization.

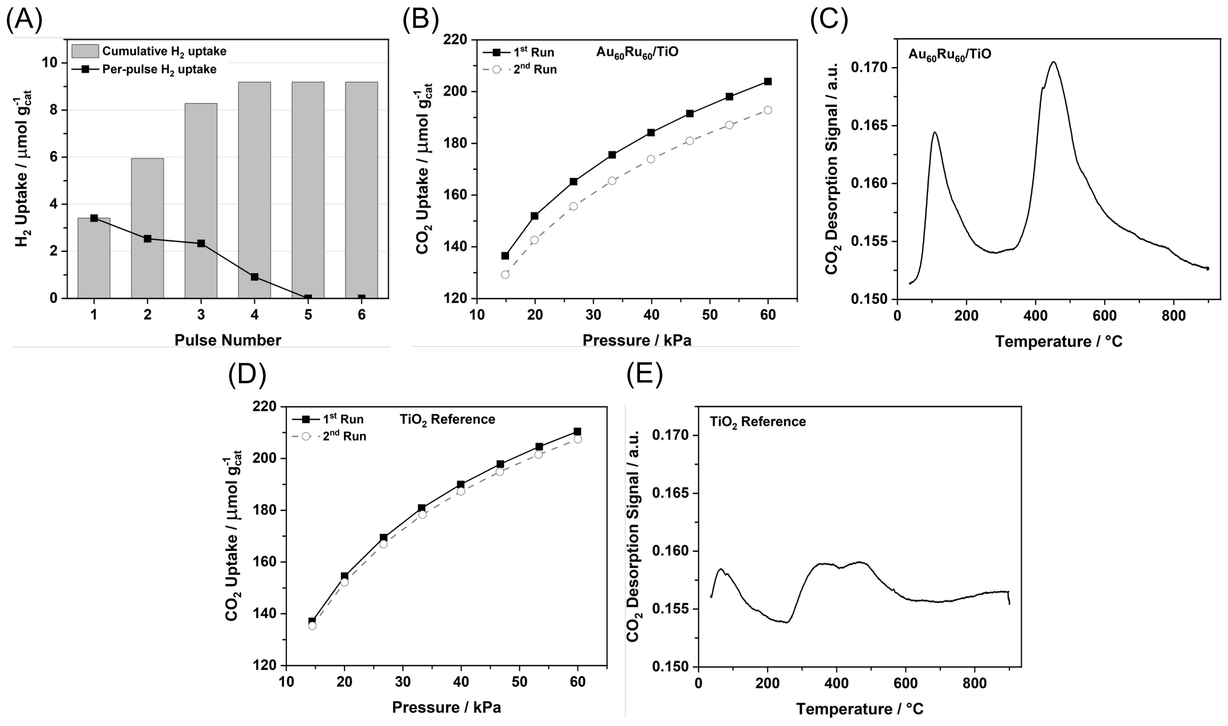

Figure S5. Surface active site characterization of the $Au_{60}Ru_{40}/TiO_2$ catalyst. (A) Pulsed H_2 chemisorption at 35 °C, used to quantify the number of accessible metallic active sites. The per-pulse uptake (squares) diminishes as the surface saturates, yielding a total cumulative uptake (bars). (B) Static CO_2 chemisorption isotherms at 35 °C. The difference between the first run (total uptake, black squares) and the second run (physisorption, open circles) is used to determine the amount of chemisorbed CO_2. (C) CO_2 TPD profile revealing multiple desorption peaks that correspond to CO_2 binding on active sites of varying strengths.

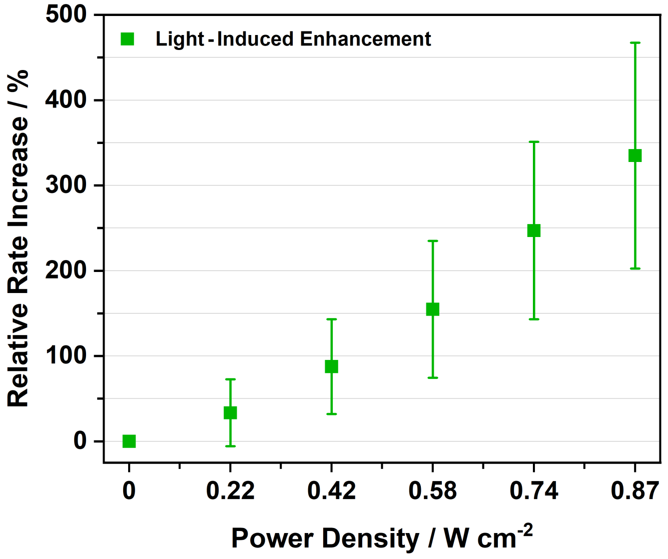

Figure S6. Light-intensity dependence of CO_2 methanation. Relative rate enhancement for the $Au_{60}Ru_{40}/TiO_2$ catalyst as a function of incident power density from a 545 nm LED at 190 °C. The enhancement, calculated relative to the dark thermal rate, increases non-linearly with light intensity.

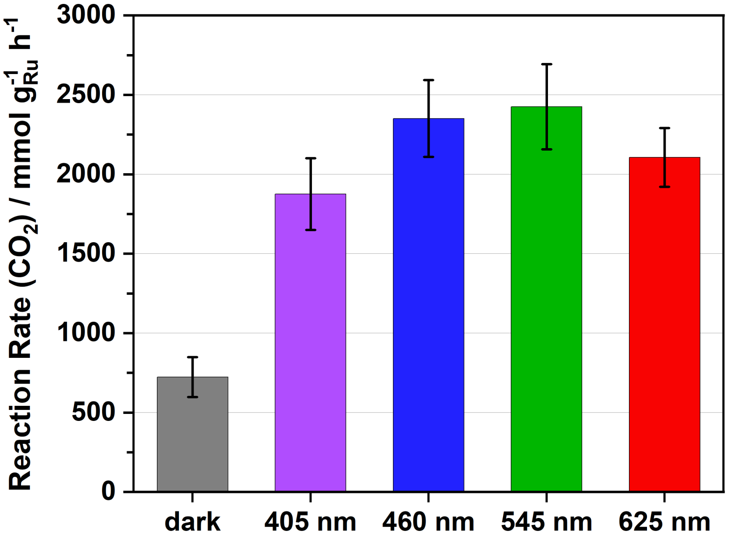

Figure S7. Wavelength- dependent plasmonic enhancement. CO_2 methanation rates for $Au_{60}Ru_{40}/TiO_2$ catalyst under different irradiation wavelengths at a constant power density (0.67 W cm^{-2}), compared to the thermal rate in the dark (grey bar). The activity profile peaks at 545 nm, correlating strongly with the catalyst's plasmon resonance and confirming the plasmon-mediated nature of the enhancement.

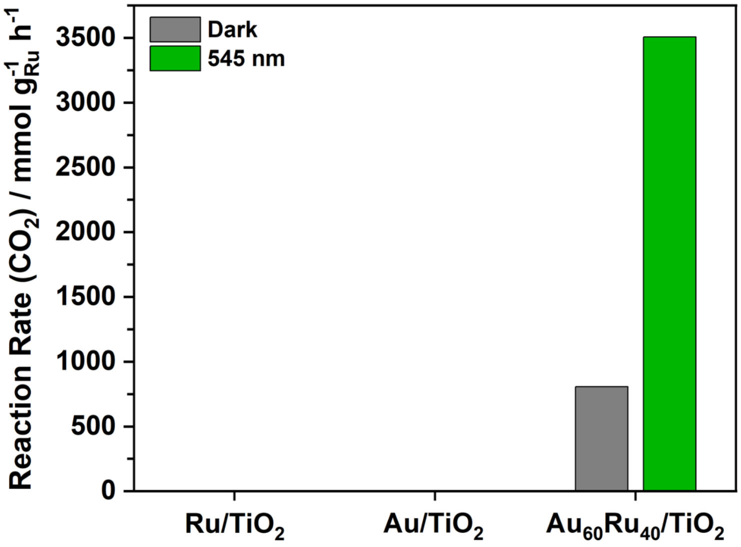

Figure S8. **Catalytic benchmarking against monometallic counterparts.** Catalytic activity for $Au_{60}Ru_{40}/TiO_2$ against their monometallic counterparts, Ru/TiO_2 and Au/TiO_2 under identical conditions (190 °C, dark and illuminated) as reported in Figure 4D, demonstrating that $Au_{60}Ru_{40}/TiO_2$ delivers higher thermal activity and a much stronger light response. Monometallic counterparts had negligible activity under both dark and illuminated conditions.

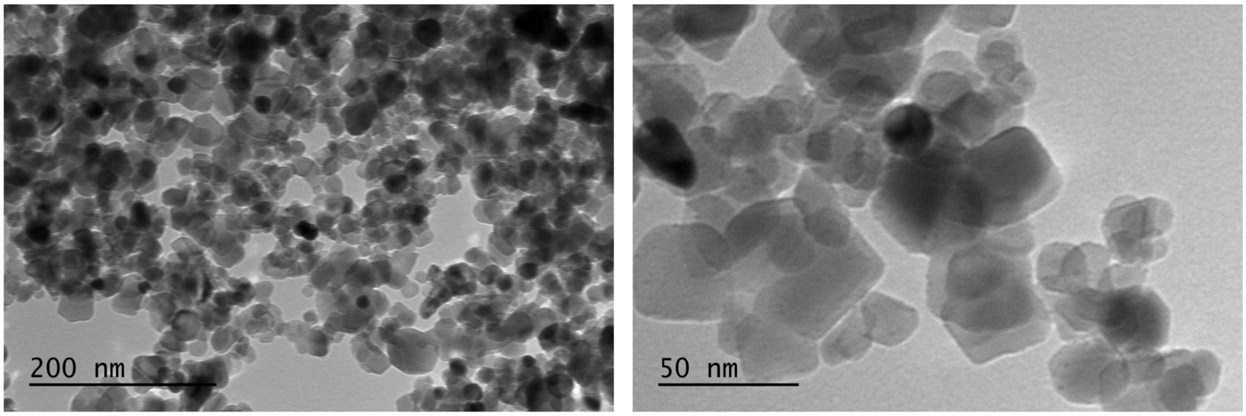

Figure S9. TEM of Au@Ru/TiO_2 after catalytic testing. Representative TEM images of $Au_{60}Ru_{40}/TiO_2$ after 85 h of continuous CO_2 methanation at 190 °C under dark conditions. The nanoparticles retain their original morphology and size distribution (Figure S3), confirming that the Au@Ru core–shell structure remains intact with no detectable sintering or aggregation. No carbonaceous deposits were observed, consistent with the excellent catalytic stability and sustained >99 % CH_4 selectivity during long-term operation.

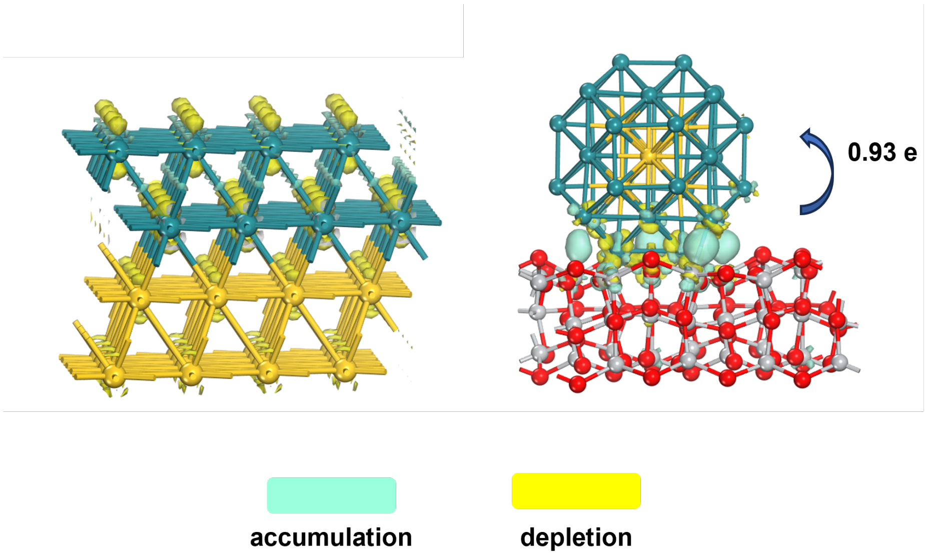

Figure S10. DFT visualization of metal-support interactions. Charge density difference plots for the (left) unsupported Au@Ru(111) and (right) Au@Ru/TiO_2(110) models. Regions of electron accumulation are shown in cyan and electron depletion in yellow (isosurface value = 0.05 e Å^{-3}). The plot for the supported catalyst reveals significant electron transfer from the TiO_2 support to the Au@Ru nanoparticle, confirming a strong metal-support interaction that modifies the electronic properties of the catalyst. Milliken charge analysis indicates a net transfer of 0.96 e^- to the nanoparticle.

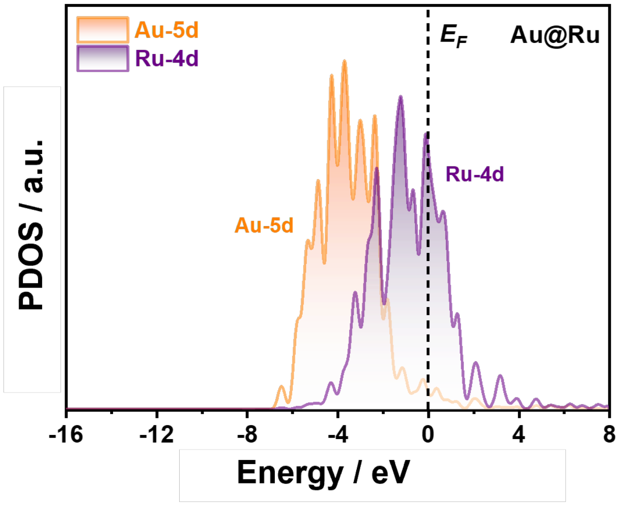

Figure S11. Electronic structure of the unsupported Au@Ru nanoparticle. Projected density of states (PDOS) calculated for the unsupported Au@Ru model, resolved for the Au 5d (orange) and Ru 4d (purple) orbitals. The analysis shows the distinct electronic contributions from the core and shell metals. The d-band center of the catalytically active Ru (dashed line) is located closer to the Fermi level (0 eV) compared to the Au core, establishing the intrinsic electronic properties of the bimetallic system before interaction with the support.

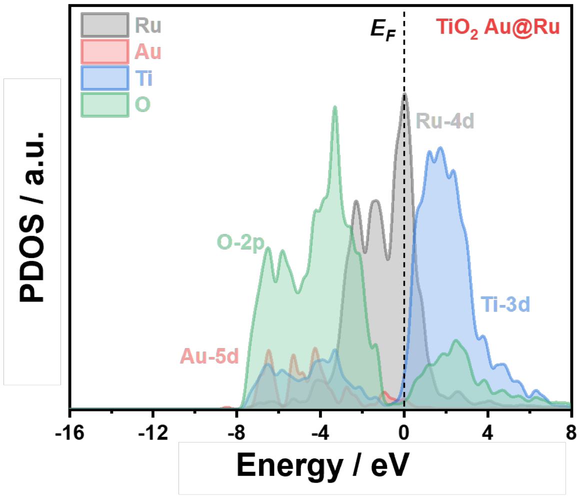

Figure S12. Electronic structure of the supported Au@Ru/TiO_2 catalyst. Projected density of states (PDOS) for the Au@Ru/TiO_2 system, resolved by element and orbital (Au 5d, Ru 4d, Ti 3d, and O 2p). The plot reveals significant hybridization between the Ru 4d states and the O 2p states of the TiO_2 support, particularly below the Fermi level (0 eV). This orbital overlap is direct evidence of the strong electronic coupling at the metal-support interface, which is responsible for modifying the catalyst's electronic properties and enhancing its activity.

Table S1. Compositional and loading analysis of Au@Ru catalysts. The table summarizes the target Au:Ru molar ratios used during synthesis, the corresponding experimental bulk compositions determined by Microwave Plasma-Atomic Emission Spectroscopy (MP-AES), and the final total Ru loading (wt.%) for each catalyst after deposition onto the TiO_2 support.

Au:Ru molar ratios employed during the synthesis	Au at. %	Ru at.%	Sample	Ru wt. % on TiO_2 supported samples
1:0.4	76	24	$Au_{76}Ru_{24}$	0.4
1:0.8	60	40	$Au_{60}Ru_{40}$	0.9
1:1.6	43	57	$Au_{43}Ru_{57}$	1.7
1:4	22	78	$Au_{22}Ru_{78}$	4.3

Table S2. Full temperature-dependent CO_2 methanation dataset for $Au_{60}Ru_{40}/TiO_2$.

T / °C	Reaction Rate (CO_2) / mmol g_{Ru}^{-1} h^{-1}				
	Dark			545 nm	
170	392	331	273	484	274
180	581	599	536	931	644
190	821	897	870	1455	1189
200	1368	1354	1366	2076	1708
210	1876	1909	1900	2865	2442
220	2609	2618	2578	3765	3210
230	3369	3341	3244	4613	4042
240	4304	4134	4054	5561	4921
250	5156	4893	4828	6520	5923
260	6107	5676	5580	7353	6856
270	6849	6319	6156	8146	7867
280	7633	6913	6815	8956	8808
290	8359	7487	7355	9688	9650
300	9037	8040	7918	10298	10458
310	9812	8484	8310	10859	11218
320	10623	8949	8697	11281	11710
330	11184	9342	9050	11642	12148
340	11692	9610	9322	11986	12542

Table S3. Dataset for Arrhenius and $E_{a,app}$ calculations. Reaction rates (dark and illuminated) and ln(r) values used to generate Arrhenius plots (Figure 3D,E) and derive temperature-dependent apparent activation energies.

***T* / K**	**1000 · T^{-1} / K^{-1}**	**Reaction Rate (CO_2) / mmol g_{Ru}^{-1} h^{-1}**		**ln(r)**	
		**Dark**	**545 nm**	**Dark**	**545 nm**
443.15	2.26	332	379	5.80	5.94
453.15	2.21	572	788	6.35	6.67
463.15	2.16	863	1322	6.76	7.19
473.15	2.11	1363	1892	7.22	7.55
483.15	2.07	1895	2654	7.55	7.88
493.15	2.03	2602	3487	7.86	8.16
503.15	1.99	3318	4328	8.11	8.37
513.15	1.95	4164	5241	8.33	8.56
523.15	1.91	4959	6221	8.51	8.74
533.15	1.88	5788	7104	8.66	8.87
543.15	1.84	6442	8007	8.77	8.99
553.15	1.81	7120	8882	8.87	9.09
563.15	1.78	7734	9669	8.95	9.18
573.15	1.74	8332	10378	9.03	9.25
583.15	1.71	8869	11039	9.09	9.31
593.15	1.69	9423	11495	9.15	9.35
603.15	1.66	9859	11895	9.20	9.38
613.15	1.63	10208	12264	9.23	9.41

Table S4. Temperature-dependent apparent activation energies for CO_2 methanation. This table provides the calculated Ea,app values and associated errors plotted in Fig. 3F. Values represent average of three runs for dark conditions and two runs for light irradiation conditions. Errors were determined by propagating the uncertainty from the ln(r) fit via numerical differentiation.

T / °C	$E_{a,app,dark}$ / kJ·mol^{-1}	$E_{a,app,545\ nm}$ / kJ·mol^{-1}
170	88.6 ± 5.5	89.7 ± 10.6
180	81.6 ± 4.6	82.2 ± 9.5
190	74.8 ± 3.7	75.1 ± 8.5
200	68.4 ± 2.8	68.3 ± 7 5
210	62.2 ± 2.0	61.7 ± 6.5
220	56.3 ± 1.2	55.5 ± 5.6
230	50.6 ± 0.4	49.4 ± 4.7
240	45.1 ± 0.3	43.6 ± 3.9
250	39.9 ± 1.0	38.1 ± 3.1
260	34.8 ± 1.7	32.7 ± 2.3
270	29.9 ± 2.4	27.5 ± 1.6
280	25.2 ± 3.0	22.6 ± 0.8
290	20.7 ± 3.6	17.8 ± 0.1
300	16.3 ± 4.2	13.1 ± 0.5
310	12.1 ± 4.8	8.7 ± 1.2
320	8.0 ± 5.3	4.3 ± 1.8
330	4.1 ± 5.9	0.2 ± 2.4
340	0.3 ± 6.4	-3.9 ± 3.0

References

[1] J. Turkevich, P. C. Stevenson, J. Hillier, "A study of the nucleation and growth processes in the synthesis of colloidal gold" *Discuss. Faraday Soc.* **1951**, *11*, 55-75.

[2] Q. Zeng, D. Liu, H. Liu, L. Xu, P. Cui, D. Chen, J. Wang, J. Yang, "Gold-catalyzed reduction of metal ions for core-shell structures with subnanometer shells" *Cell Rep. Phys. Sci.* **2022**, *3*, 101105.

[3] J. P. Perdew, K. Burke, M. Ernzerhof, "Generalized Gradient Approximation Made Simple" *Phys. Rev. Lett.* **1996**, *77*, 3865-3868.

[4] A. A. Peterson, F. Abild-Pedersen, F. Studt, J. Rossmeisl, J. K. Nørskov, "How copper catalyzes the electroreduction of carbon dioxide into hydrocarbon fuels" *Energy Environ. Sci.* **2010**, *3*, 1311-1315.

[5] P. B. Johnson, R. W. Christy, "Optical Constants of the Noble Metals" *Phys. Rev. B* **1972**, *6*, 4370-4379.

[6] D. L. Windt, W. C. Cash, M. Scott, P. Arendt, B. Newnam, R. F. Fisher, A. B. Swartzlander, "Optical constants for thin films of Ti, Zr, Nb, Mo, Ru, Rh, Pd, Ag, Hf, Ta, W, Re, Ir, Os, Pt, and Au from 24 Å to 1216 Å" *Appl. Opt.* **1988**, *27*, 246-278.

[7] J. Feser, A. Sobh, **2016**.

[8] B. O. Community, "Blender-a 3D modelling and rendering package" *Blender Foundation* **2018**.

[9] B. T. Draine, P. J. Flatau, "Discrete-Dipole Approximation For Scattering Calculations" *J. Opt. Soc. Am. A* **1994**, *11*, 1491-1499.

[10] J. J. Goodman, B. T. Draine, P. J. Flatau, "Application of fast-Fourier-transform techniques to the discrete-dipole approximation" *Opt. Lett.* **1991**, *16*, 1198-1200.

[11] M. J. Collinge, B. T. Draine, "Discrete-dipole approximation with polarizabilities that account for both finite wavelength and target geometry" *J. Opt. Soc. Am. A* **2004**, *21*, 2023-2028.

[12] B. T. Draine, J. Goodman, "Beyond Clausius-Mossotti: Wave Propagation on a Polarizable Point Lattice and the Discrete Dipole Approximation" *Astrophys. J.* **1993**, *405*, 685.

[13] B. T. Draine, P. J. Flatau, "User guide for the discrete dipole approximation code DDSCAT 7.3" *arXiv preprint arXiv:1305.6497* **2013**.
